# Regulation of platelet-activating factor-mediated interleukin-6 promoter activation by the 48 kDa but not the 45 kDa isoform of protein tyrosine phosphatase non-receptor type 2

**DOI:** 10.1186/s13578-019-0316-9

**Published:** 2019-06-25

**Authors:** Geneviève Hamel-Côté, Fanny Lapointe, Steeve Véronneau, Marian Mayhue, Marek Rola-Pleszczynski, Jana Stankova

**Affiliations:** Immunology Division, Department of Pediatrics, Faculty of Medicine and Health Sciences, Université de Sherbrooke, Sherbrooke, QC Canada

**Keywords:** Platelet-activating factor, GPCR, Protein tyrosine phosphatase, IL-6, TC-PTP, PTPN2

## Abstract

**Background:**

An underlying state of inflammation is thought to be an important cause of cardiovascular disease. Among cells involved in the early steps of atherosclerosis, monocyte-derived dendritic cells (Mo-DCs) respond to inflammatory stimuli, including platelet-activating factor (PAF), by the induction of various cytokines, such as interleukin 6 (IL-6). PAF is a potent phospholipid mediator involved in both the onset and progression of atherosclerosis. It mediates its effects by binding to its cognate G-protein coupled receptor, PAFR. Activation of PAFR-induced signaling pathways is tightly coordinated to ensure specific cell responses.

**Results:**

Here, we report that PAF stimulated the phosphatase activity of both the 45 and 48 kDa isoforms of the protein tyrosine phosphatase non-receptor type 2 (PTPN2). However, we found that only the 48 kDa PTPN2 isoform has a role in PAFR-induced signal transduction, leading to activation of the IL-6 promoter. In luciferase reporter assays, expression of the 48 kDa, but not the 45 kDa, PTPN2 isoform increased human IL-6 (hIL-6) promoter activity by 40% after PAF stimulation of HEK-293 cells, stably transfected with PAFR (HEK-PAFR). Our results suggest that the differential localization of the PTPN2 isoforms and the differences in PAF-induced phosphatase activation may contribute to the divergent modulation of PAF-induced IL-6 promoter activation. The involvement of PTPN2 in PAF-induced IL-6 expression was confirmed in immature Mo-DCs (iMo-DCs), using siRNAs targeting the two isoforms of PTPN2, where siRNAs against the 48 kDa PTPN2 significantly inhibited PAF-stimulated IL-6 mRNA expression. Pharmacological inhibition of several signaling pathways suggested a role for PTPN2 in early signaling events. Results obtained by Western blot confirmed that PTPN2 increased the activation of the PI3K/Akt pathway via the modulation of protein kinase D (PKD) activity. WT PKD expression counteracted the effect of PTPN2 on PAF-induced IL-6 promoter transactivation and phosphorylation of Akt. Using siRNAs targeting the individual isoforms of PTPN2, we confirmed that these pathways were also active in iMo-DCs.

**Conclusion:**

Taken together, our data suggest that PTPN2, in an isoform-specific manner, could be involved in the positive regulation of PI3K/Akt activation, via the modulation of PKD activity, allowing for the maximal induction of PAF-stimulated IL-6 mRNA expression.

**Electronic supplementary material:**

The online version of this article (10.1186/s13578-019-0316-9) contains supplementary material, which is available to authorized users.

## Introduction

Chronic inflammation is characterized by the continuous activation of signaling pathways involved in cell survival and promotion of leukocyte recruitment, associated with angiogenesis and reactive-oxygen species (ROS) production, all linked to the progression of atherosclerosis [1, 2]. Produced by oxidized lipid-injured endothelium, PAF is found early in atherosclerosis onset and is involved in many processes leading to the progression of the plaque such as migration, adhesion and cytokine and chemokine production by a myriad of cell types [3, 4]. PAF activity is mediated by binding to its cognate G-protein coupled receptor, PAFR [5]. PAFR is expressed in a wide assortment of cells involved in atherosclerosis, from leucocytes such as neutrophils, macrophages, dendritic cells and monocytes to smooth muscles cells and endothelial cells [6, 7]. This widespread receptor expression could explain why PAF would be involved at numerous stages of this disease.

Among the cells found early in the onset of the atherosclerotic lesion, immature monocyte-derived dendritic cells (iMo-DCs) could be one of the first cells to respond to PAF produced by activated endothelial cells. In fact, in rodents, monocytes are recruited to atherosclerotic risk zones where they contribute to the increase in dendritic cell numbers [8, 9]. Due to their lower expression of PAF acetyl-hydrolase, these cells are more sensitive to PAF than monocyte-derived macrophages, one of the best-characterized contributors to atherosclerosis progression [10]. Hence, iMo-DCs could respond earlier and to lower PAF concentrations than monocyte-derived macrophages, by secreting cytokines and other mediators. PAF is involved in the induction of many pro-inflammatory and growth factors; among them, interleukin-6 (IL-6) is one of the most interesting, in view of its role in atherosclerosis. A moderate, but sustained, increase in circulating IL-6 levels correlates with increased risk of developing coronary heart disease [11]. Previous studies have shown that PAF stimulates IL-6 production by endothelial cells, alveolar and peritoneal macrophages and smooth muscle cells [12–15]. In smooth muscle cells, IL-6 production, stimulated by PAF, depends on protein tyrosine kinase (PTK) activation [14]. Among PTKs activated by PAF are FAK (Focal Adhesion Kinase), Src, Jak2 and Tyk2 [16–18]. In the MonoMac1 cell line, activated Jak2 and Tyk2 lead to phosphorylation and activation of STAT1, 2, 3 and 5 [17], whereas in HUVECs (Human umbilical vein endothelial cells), Src has been shown to contribute to STAT3 activation [16]. Interestingly, STAT1 and STAT5 have been shown to be, directly or indirectly, involved in IL-6 production, either via the induction of transcription factors such as IRF-1 that can directly bind the IL-6 promoter or via their interaction with other transcription factors such as p50-NFκB [19–24]. Many of the kinases and their substrates, mentioned above, are also substrates of protein tyrosine phosphatase non-membrane type 2 (PTPN2, also named TCPTP (T-cell protein tyrosine phosphatase or PTP-S in rat) [25]. PTPN2 is expressed as two isoforms of 45 and 48 kDa, generated by alternative splicing in human and rat cells, but only the 45 kDa isoform is found in murine cells [26, 27]. Ratios of the isoforms differ between cell types: a higher quantity of 48 kDa is found in the Jurkat T cell line and the THP-1 myeloid cell line whereas the 45 kDa isoform comprises the majority of PTPN2 expressed in the human hepatocyte cell line HepG2 and human melanoma cell lines A375 and M14 [28]. Despite many shared substrates such as the insulin receptor, EGFR and TRAF2 [29, 30], the two isoforms differ in many ways. First, they are localized in different sub-cellular compartments: the 45 kDa variant is targeted to the nucleus by a regulated mechanism of transport involving interaction with p97 and its bi-partite nuclear localization sequence (NLS) [31]. The 48 kDa isoform is confined to the endoplasmic reticulum (ER) by its interaction with p25, a member of the p24 family involved in retro-transport from the Golgi to the ER [32]. Second, they have different substrates: the 45 kDa isoform, but not the 48 kDa one, dephosphorylates nuclear STAT6 [33] and interacts with p52Shc, impairing p42 Erk phosphorylation [30]. On the other hand, only the 48 kDa isoform is involved in anchorage-independent cell growth, probably acting on pathways important for cyclin D1 expression [34]. Third, they are differentially regulated: the 45 kDa, but not the 48 kDa, variant is phosphorylated during mitosis by CDK [35]. In addition, only the 45 kDa isoform sees its localization changed during stimulation: it can shuttle out of the nucleus during cell stress to interact with its cytoplasmic substrates [31, 36]. The fact that their localization is tightly regulated, combined with the differences in regulatory mechanisms and substrate specificity, supports the idea that each isoform has a different cellular function, making them an interesting target for study in pathways activated by PAFR. In this report, we studied the involvement of both PTPN2 isoforms in IL-6 transcriptional activation by PAFR.

## Results and discussion

### Differential effects of PTPN2 isoforms on PAF-induced hIL-6 promoter activity

**Fig. 1:**
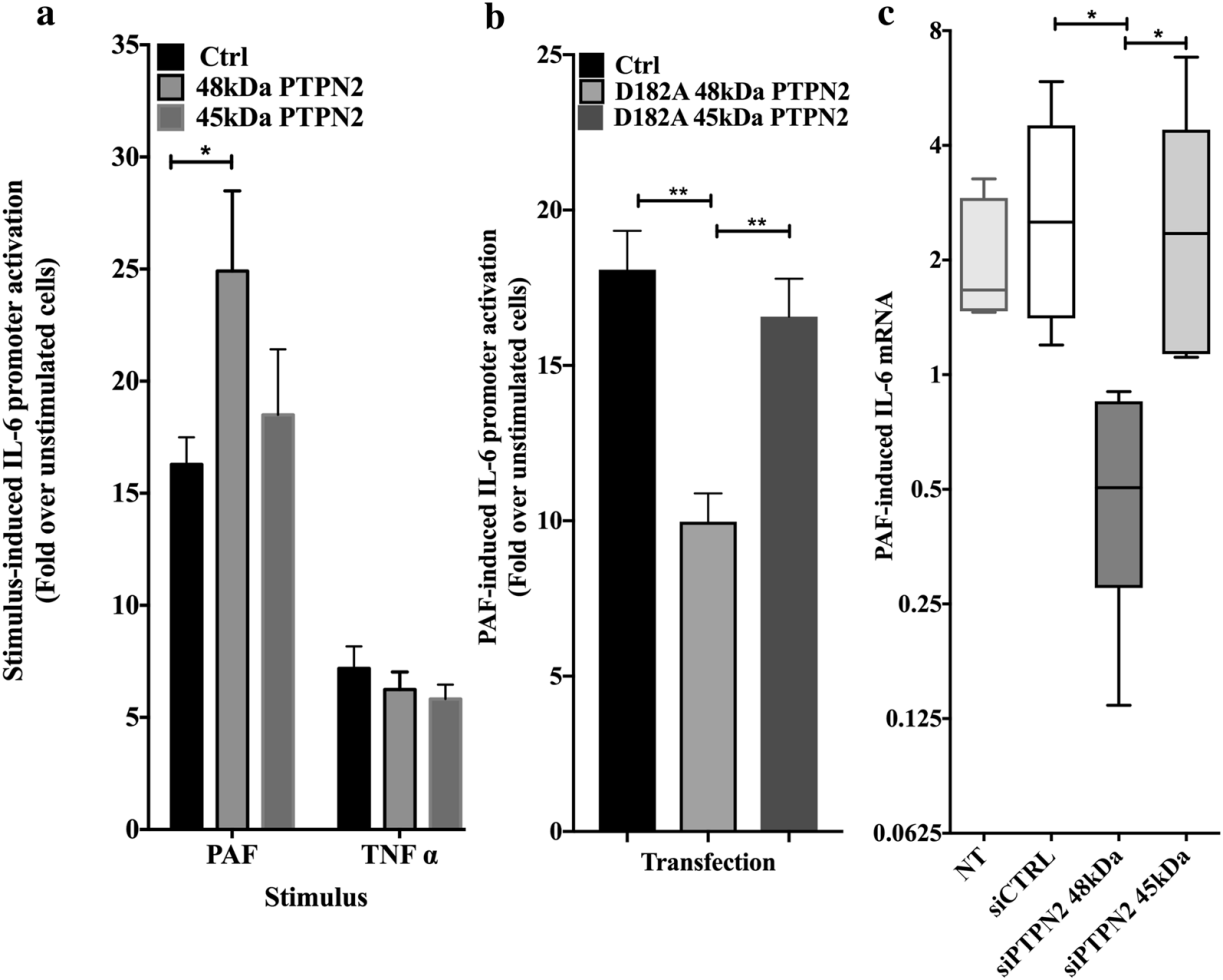
Differential effects of PTPN2 isoforms on PAF-induced IL-6 expression. **a** HEK-PAFR were transiently co-transfected with the hIL-6-luc, control vector (pcDNA3) or phosphatase constructs **a** WT and **b** D182A mutants. Cells were incubated overnight in DMEM-0.2% BSA and stimulated with **a**, **b** PAF (100 nM), vehicle or **a** TNF-α (20 ng/ml) for 6 h and luciferase activity was measured. The data presented are a mean ± SEM of 6–16 independent experiments performed in triplicate. Significance was established with two-way ANOVA with Sidak post-test. *p < 0.05. **c** iMo-DCs were either not transfected (NT) or transfected with siRNAs: either control (siCTRL) or against isoforms of PTPN2 (siPTPN2 48 kDa or 45 kDa) on day 4 and 5 before being collected on day 7 for experiments. iMo-DCs were starved for 3 h in RPMI 0.2% BSA and stimulated with PAF (1 nM) for 5 h. Cells were lysed in Trizol and RNA was extracted and converted to cDNA. IL-6 mRNA was quantified by real-time PCR. Data are presented as mean ± SEM of mRNA expression calculated by the delta–delta (ΔΔ)Ct method on their respective unstimulated control for 4 independent experiments, using RPL13A and GAPDH as housekeeping genes. Significance was established with two-way ANOVA with Sidak post-test, *p < 0.05

Previous reports have shown a PAF-stimulated increase of IL-6 mRNA and protein expression in a variety of cell types [12, 13, 37]. To investigate regulation of PAF-induced IL-6 expression, we concentrated on PTPN2, in order to determine whether one, or both, isoforms of PTPN2 were involved in regulation of IL-6 promoter activation induced by PAF. First, HEK-293 cells, stably expressing the HA-tagged PAFR (HEK-PAFR), were transfected with the human IL-6 promoter coupled to the luciferase gene (hIL-6-luc) and with human PTPN2 constructs coding for the 45 or 48 kDa isoforms. Thirty-six hours post-transfection, cells were stimulated with PAF or TNF-α (Tumor necrosis factor alpha). As shown in Fig. 1a, PAF-induced activation of hIL-6 was significantly higher when the 48 kDa isoform of PTPN2 was co-expressed, compared to cells transfected with an empty vector or the 45 kDa PTPN2 isoform. In addition, co-expression of either the 48 kDa or the 45 kDa isoform did not modulate the activation of the promoter by TNF-α, indicating that the up-regulation of IL-6 expression by 48 kDa PTPN2 could be agonist-specific. In contrast, Van Vliet et al. have shown that the 45 kDa isoform modulates TNFα-induced IL-6 production in reconstituted MEF (murine embryonic fibroblasts) [29]. The differences between the two studies could be explained by our use of human cells which express both isoforms, whereas the murine MEF normally express only the 45 kDa variant and by the differences between both stimuli in regards of signaling pathways involved in IL-6 promoter activation. Moreover, the phosphatase activity of the 48 kDa isoform of PTPN2 seems to be required for the increase of IL-6 expression induced by PAF treatment given that transfection of the D182A mutant, which has a lower K_cat_ [38], significantly decreased the transactivation of the IL-6 promoter in HEK-PAFR, whereas the same mutation of the 45 kDa isoform did not modulate PAF-induced IL-6 promoter activity (Fig. 1b).

The importance of PTPN2 in PAF-induced signalization was also seen in iMo-DCs, cells that are key in the pro-inflammatory context of atherosclerosis. PAF-induced IL-6 mRNA levels in iMo-DCs were measured by RT-PCR. PAF induced an approximately twofold increase in IL-6 mRNA in untransfected iMo-DC, siCTRL- and siPTPN2 45 kDa-transfected cells. In contrast, PAF induced significantly less IL-6 mRNA in siPTPN2 48 kDa-transfected cells than in siCTRL- and siPTPN2 45 kDa-transfected cells, suggesting that the 48 kDa isoform of PTPN2 was important for the increase of IL-6 mRNA levels when cells were stimulated with PAF (Fig. 1c).

The specificity of our siRNAs was confirmed by RT-PCR. The results of the quantification by the delta–delta (ΔΔ)Ct method was further confirmed by PCR given the use of DMSO in the RT-PCR mix, which could affect RT-PCR reactions. DMSO was added due to the formation of secondary structure caused by A/T repeated sequences in the amplified regions of PTPN2. The identity of amplified fragments was confirmed by sequencing. siRNAs against the 45 kDa isoform of PTPN2 (siPTPN2 45 kDa) caused a significant decrease, approximately 30–40%, in the 45 kDa isoform without appreciably affecting the 48 kDa. The siRNAs against the 48 kDa isoform of PTPN2 (siPTPN2 48 kDa) showed the same specificity when compared to siCTRL (Additional file 1: Fig. S1A–C). Protein levels were also monitored and a consequent decrease was observed after transfection with siRNAs. Nevertheless, these results should be taken with caution, given the close molecular weights of both isoforms and the possible post-translational modifications (Additional file 1: Fig. S1D–F). Neither the level of the highly related PTPN1 nor the cell mortality were affected by the siRNAs, indicating their specificity (Additional file 1: Fig. S1G–I).

### The isoform-specific effects of PTPN2 may be due to differences in localization and in long-term phosphatase activity

We examined mechanisms that could explain the observed differences in PTPN2 isoform effects. First, we monitored the PTP protein expression levels. For this, HEK-PAFR were transfected with GFP-48 kDa or GFP-45 kDa PTPN2 constructs, using the same transfection conditions as ones used for the luciferase assays and expression levels were followed by flow cytometry. Results showed that a similar percentage of cells expressed both of the PTPN2 isoforms (Additional file 1: Fig. S1J). However, higher geo means found in GFP-45 kDa PTPN2-transfected cells than in GFP-48 kDa PTPN2-transfected cells indicated a higher expression levels per cell for the 45 kDa isoform (Additional file 1: Fig. S1K), reducing the likelihood that the absence of modulation of PAF-induced IL-6 promoter, by this isoform, was due to lower expression levels. PTPN2 isoforms did not modulate IL-6 promoter activation in unstimulated cells (Additional file 1: Fig. S1L).

**Fig. 2:**
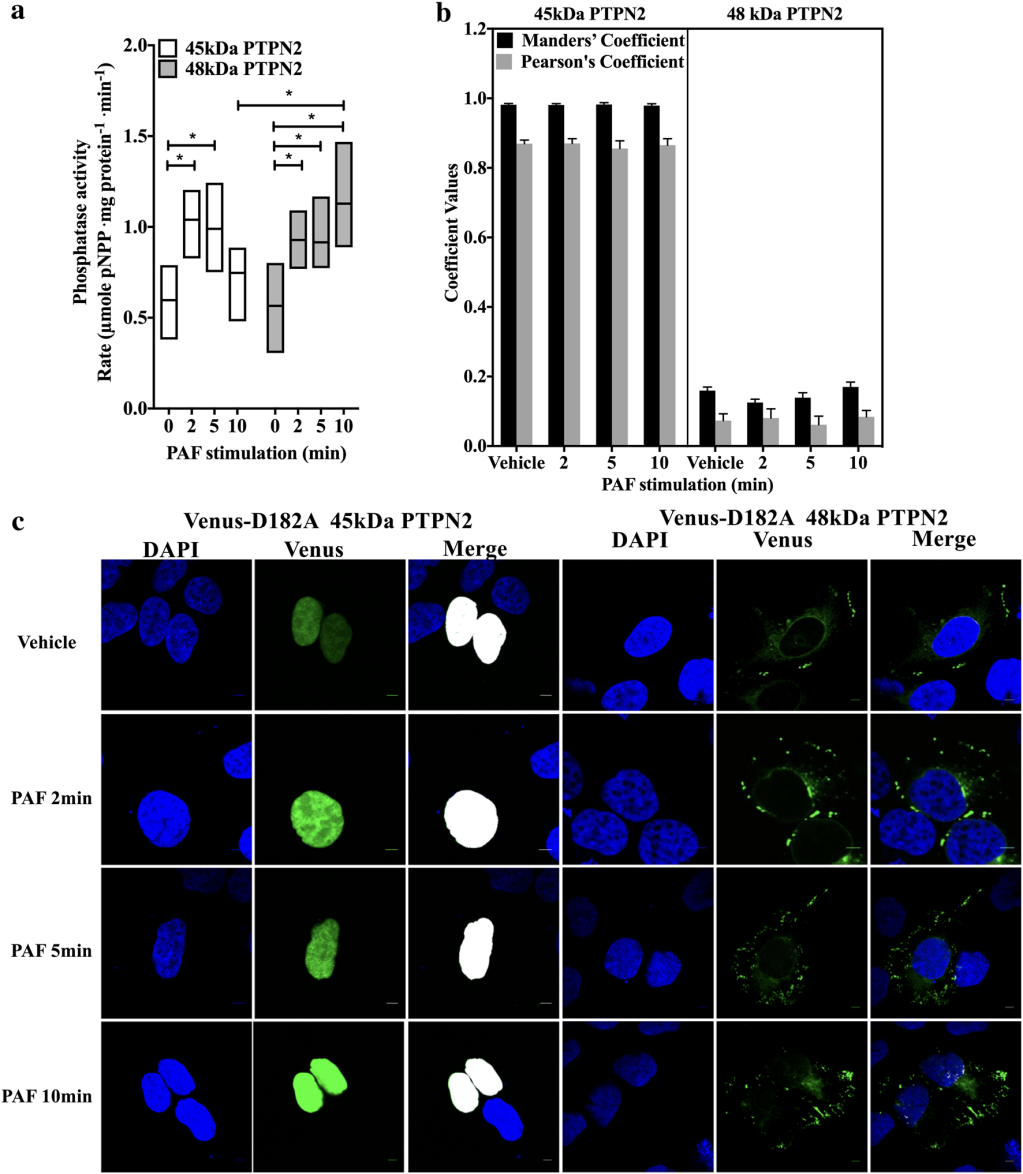
PAF modulates 45 kDa and 48 kDa PTPN2 activity but not the 45 kDa PTPN2 localization. **a** Graph represents the phosphatase activity determined by the hydrolysis rate of pNPP by Flag-tagged PTPN2, immunoprecipitated from HEK-PAFR which had been starved overnight in DMEM 0.2% BSA before being stimulated with 100 nM PAF, for indicated times. After PTP experiments, proteins were separated by SDS-PAGE, transferred to nitrocellulose membranes and blotted overnight with anti-PTPN2 antibodies for normalization of pNPP rates. Data are presented as low–high graphs (with lines at mean) of normalized pNPP hydrolysis rates for 3 independent experiments. Significance was established with paired two-way ANOVA with Sidak post-test. *p < 0.05. **b**, **c** HEK-PAFR, grown on poly-l-lysine coated coverslips, were transfected with Venus-tagged D182A-PTPN2 then stimulated, 30 h later, with vehicle or 100 nM PAF for 2, 5 or 10 min before permeabilization with 0.5% Triton X-100. Nuclei were stained with DAPI and cells were analyzed by confocal microscopy. Results are representative of 4 independent experiments, with at least 28 pictures analyzed from different fields per condition. Scale bar = 5 µm. **b** Compilation of Pearsons’ and Manders’ Coefficients for PTPN2. Data presented are mean ± SEM of 4 independent experiments and **c** representative pictures of Venus-tagged D182A 45 kDa or 48 kDa-D182A PTPN2 isoforms

Next, we considered the possibility that the differences found between PTPN2 isoform effects could be due to a difference in phosphatase activity. To test this, phosphatase assays were performed with Flag-tagged 45 kDa or − 48 kDa PTPN2 immunoprecipitated from HEK-PAFR lysates. Western blots were done with the immunoprecipitates to ensure that a similar amount of protein was used (Additional file 1: Fig. S2A). First, the specificity of the assay, was tested by transfected Flag-tagged WT or D182A-PTPN2 isoforms in HEK-PAFR. Mutation of this aspartate, which is conserved among PTPs, results in an important decrease in Kcat without affecting affinity, producing a trapping mutant [30, 38]. As shown in Additional file 1: Fig. S2, only WT isoforms had pNPP hydrolysis rates that differed significantly from background. When normalized with immunoprecipitated protein levels, both WT isoforms had a similar phosphatase activity levels in vehicle stimulated cells (Fig. 2a, t = 0) or in unstimulated cells (Additional file 1: Fig S2B). This is consistent with data found in literature showing that both isoforms have a similar hydrolysis rate for small substrates such as pNPP (p-nitrophenyl phosphate) [39] and also suggests that there is no difference in basal regulation between these isoforms. Importantly, PAF increased the phosphatase activity levels of both PTPN2 isoforms, although the activation of the 45 kDa one was transient and decreased after 5 min of stimulation, whereas, the 48 kDa isoform phosphatase activity levels remained significantly higher at 10 min of stimulation. These results are consistent with our observations showing that PAF can modulate the activity of other phosphatases by increasing their expression [40] or their enzymatic activity [41]. In addition, others have shown that the catalytic activity of the 45 kDa isoform or both isoforms without distinction, is increased by stimuli such as IFNγ [42], adhesion [43] and VEGFR [44]. However, this is, to our knowledge, the first time that a direct assessment of the 48 kDa PTPN2 catalytic activity, alone, shows an increase by a cellular stimulus. Mechanisms by which PAF induced this increase remain to be elucidated and, apart from oxidation [45, 46], little is known about how cells modulate PTPN2 catalytic activity. Indeed, post-translational modifications such as serine phosphorylation by CDK [35] or PKR [47] modulate its ability to dephosphorylate its substrates in vivo without affecting the catalytic activity. On the other hand, the phosphatase activity levels of the 45 kDa isoform have been shown to be lowered by an intramolecular interaction between the non-catalytic C-terminal domain and the PTP domain; an interaction that can be disrupted by the binding of proteins, such as the cytoplasmic tail of the α-1 integrin, or compounds, such as the chemotherapeutic agent mitoxantrone [43, 44, 48, 49]. The presence of such an intramolecular interaction in the 48 kDa isoform and the possible impacts of interacting proteins such as C3G or syntaxin 17 on its phosphatase activity are still unknown [50–52]. Further investigation will be needed, but these results suggest that variations in the kinetics of PTPN2 isoform activity could account for at least some of the differences in the modulation of PAF-induced IL-6 promoter activation.

Another important mechanism that has been involved in the isoform specific effect of PTPN2 is the difference in cellular localization. For example, the 45 kDa isoform is involved in nuclear de-phosphorylation of STAT6 (pSTAT6), its nuclear localization excluding a role for the 48 kDa isoform in this process [33]. Therefore, we determined PTPN2 intracellular localization by using Venus D182A-PTPN2-transfected HEK-PAFR and the nucleus as a reference point, given that stimuli such as IFNγ [42] or EGF [53] can induce the exit of the 45 kDa isoform from the nucleus. As the D182A mutation greatly slow down the catalytic reaction without affecting the substrate specificity [38], substrate trapping mutants were used to stabilize any possible interaction between the PTPN2 phosphatase domain and substrates and thereby, the localization. Results obtained suggest that the 45 kDa isoform had a nuclear location in vehicle-stimulated cells (Fig. 2b, c) and this was not changed by PAF stimulation (Manders’ Coefficients > 0.974 and Pearson’s Coefficient > 0.85, n > 28). On the other hand, consistent with data from literature, the 48 kDa-D182 isoform did not significantly colocalize with the nucleus (Manders’ Coefficients < 0.17 and Pearson’s Coefficient < 0.08, n > 28) but was rather found in the perinuclear and cytoplasmic areas, both in vehicle- and PAF-stimulated cells. Accumulation of PTPN2 aggregates were also found at the cellular periphery (Fig. 2b, c) suggesting that this isoform may interact with substrates at this location, as described previously by others for PTP1B [54, 55]. Altogether, these results suggest that both, the differential localization of the PTPN2 isoforms and the differences in PAF-induced phosphatase activation may contribute to the divergent modulation of PAF-induced IL-6 promoter activation.

### Which PAF-stimulated pathways are modulated by PTPN2 48 kDa?

#### MAPKs, PKC and SFK pathways

**Fig. 3:**
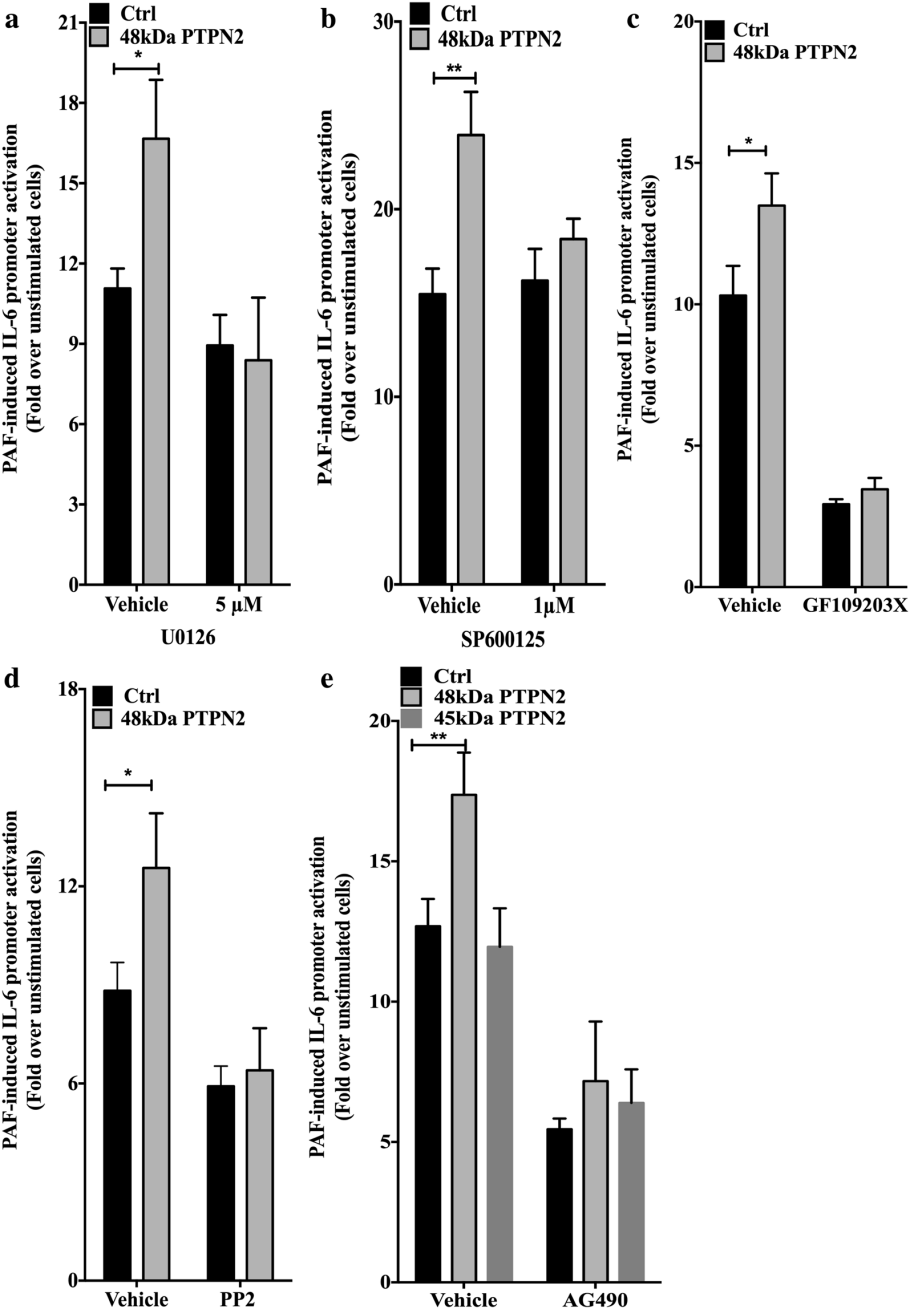
Modulation of PAF-induced IL-6 promoter activity by 48 kDa PTPN2 depends on multiple signaling pathways. HEK-PAFR were transiently co-transfected with the hIL-6-luc, control vector (pcDNA3), or the 48 kDa or 45 kDa PTPN2 constructs. Cells were incubated overnight in DMEM-0.2% BSA and **a**–**e** pre-treated for 30 min in DMEM-0.2%BSA with DMSO as vehicle control or **a** MEK 1/2 inhibitor U-0126, 5 µM, **b** JNK inhibitor SP600125, 1 µM, **c** pan PKC inhibitor GF109203x 2 µM or **d** Src kinase family inhibitor PP2, 10 nM and **e** Jak2 inhibitor AG490 5 µM. Stimulation with PAF (100 nM) or vehicle was for 6 h and luciferase activity was then measured. The data presented are a mean ± SEM of at least 4 independent experiments performed in triplicate. Significance was established with two-way ANOVA with Sidak post-test. *p < 0.05, **p < 0.01

We next investigated the signaling pathways stimulated by PAF and potentially affected by PTPN2. First, we examined the MAPK pathways involved in IL-6 transcriptional activation by PAF [29, 56], initially investigating the MEK/Erk pathway. Both G-protein-dependent and -independent pathways [5, 57] are involved in the activation by PAF of the MEK/Erk pathway which is able to modulate activity of AP-1 [58] C/EBPß [59], STAT5 [17] and NFκB [60], all transcription factors involved in IL-6 promoter activation [62]. Control- or 48 kDa PTPN2-transfected HEK-PAFR cells were pre-incubated with U0126, a MEK1/2 inhibitor, and IL-6 promoter activity was evaluated after stimulation with PAF. MEK inhibition caused a small decrease in PAF-stimulated hIL-6 promoter activation in control cells, but completely abrogated the increase seen in PTPN2-transfected cells, suggesting the participation of this pathway in PTPN2-initiated potentiation of IL-6 activation (Fig. 3a). We confirmed that PTPN2 was involved in the increase in PAF-induced phosphorylation of Erk1/2, a downstream target of MEK1 and 2, by performing Western blots with lysates obtained from HEK-PAFR transfected with control vector or either isoform of PTPN2, then stimulated with PAF for indicated times. As expected from the luciferase assays, the 48 kDa isoform but not the 45 kDa isoform increased the phosphorylation of Erk (Additional file 1: Fig. S3A, B). These results are consistent with those from other studies showing that PTPN2 regulates ERK 1 and 2 phosphorylation [29]. An inhibitor of the JNK MAPK pathway, JNK inhibitor II, also decreased the PTPN2-mediated increase in PAF-induced hIL-6 promoter activation, while control cell activity was not modified (Fig. 3b).

PKC and Src are both kinases that can be found upstream of MAPKs [61, 62]. We tested the involvement of PKCs, a family of serine/threonine kinases, which have been shown to be activated early in PAF-induced signaling pathways, using the pan-PKC inhibitor GF109203X. PKC inhibition drastically reduced the PAF-induced transcription of IL-6 and also eliminated the PTPN2-dependent increase (Fig. 3c). However, due to the essential role of these kinases in the transcriptional activation of IL-6 by PAF, it is difficult to reach a firm conclusion on their participation in PTPN2-dependent increase in IL-6 promoter transactivation. To evaluate whether Src kinases contributed to this activity, we used PP2, a selective Src family kinase (SFK) inhibitor. Pre-treatment with 10 nM PP2 induced a significant decrease in IL-6 promoter transactivation in control cells (Fig. 3d), suggesting a role of the SFK in the PAF-induced transactivation of IL-6 promoter, consistent with its involvement in the modulation of several proteins involved in IL-6 promoter transactivation such as STAT3 [63], NFκB [64] and ERK [16, 56]. Moreover, this SFK inhibitor abrogated the 48 kDa PTPN2-mediated increase in PAF-induced IL-6 promoter which indicated that the 48 kDa PTPN2 could be involved in the upregulation of SFK activity (Fig. 3d). Consistent with this, when the PTPN2 D182A substrate-trapping mutants were transfected in HEK-PAFR, the 48 kDa, but not the 45 kDa, D182A isoform decreased the phosphorylation of SFK on Y419, the tyrosine in the activation loop the phosphorylation levels of which correlate with SFK activation levels (Additional file 1: Fig. S3C, D). Moreover, the 48 kDa, but not the 45 kDa, mutant isoform, significantly decreased PAF-induced IL-6 transactivation in a manner that seemed to involve the SFKs, given that inhibition of these kinases did not result in any further modulation of promoter transactivation in D182A 48 kDa PTPN2-transfected cells whereas it significantly decreased it in control or D182A 45 kDa PTPN2-transfected cells. These results are surprising given that PTPN2 is known as a negative regulator of Src, binding and dephosphorylating it on the Y419 residue [29, 65] and given that the substrate-trapping mutants of PTPN2 form, when directly binding to pY419-SFKs, a stable complex, enhancing SFK phosphorylation levels by protecting the targeted phospho-tyrosine from dephosphorylation [66]. Therefore, these results suggest that even if PTPN2 increased PAF-induced SFK activation, SFKs were not primary targets of PTPN2.

Altogether, these results indicate that the PTPN2-mediated increase in IL-6 promoter activity may be dependent on the majority of tested pathways, suggesting that PTPN2 plays a role very early in PAFR signaling.

#### Early signaling pathways: G-proteins and Jak2

PAF induces signaling via both G-protein-dependent and -independent pathways. The G-protein-independent pathways are via arrestin or Tyk2/Jak2 [17, 18, 57, 60, 67]. We had also shown that PAF activates STAT 1, 3 and 5 in a Tyk2-dependent manner [17] and that Jak2 activation could counteract the Tyk2-dependent transactivation of PAFR promoter [18]. We therefore examined whether PTPN2 could modulate the PAF-induced IL-6 through a Jak2-dependent pathway. We included the 45 kDa isoform as a control, given that all three STAT proteins are potential substrates for this isoform and all can potentially transactivate the IL-6 promoter alone or in synergy with other transcription factors [19, 22–24, 68]. HEK-PAFR cells were transfected with the 48 kDa or the 45 kDa isoform of PTPN2, or their controls, together with the hIL-6 promoter coupled to luciferase and then incubated with the Jak2 inhibitor AG490 (Fig. 3e). Results showed that Jak2 activity was involved in PAF-induced IL-6 promoter activation and in 48 kDa PTPN2-mediated increase in hIL-6 promoter activity, suggesting that Jak2 downstream effectors were also involved, at least partially, in PTPN2-mediated IL-6 promoter transactivation.

**Fig. 4:**
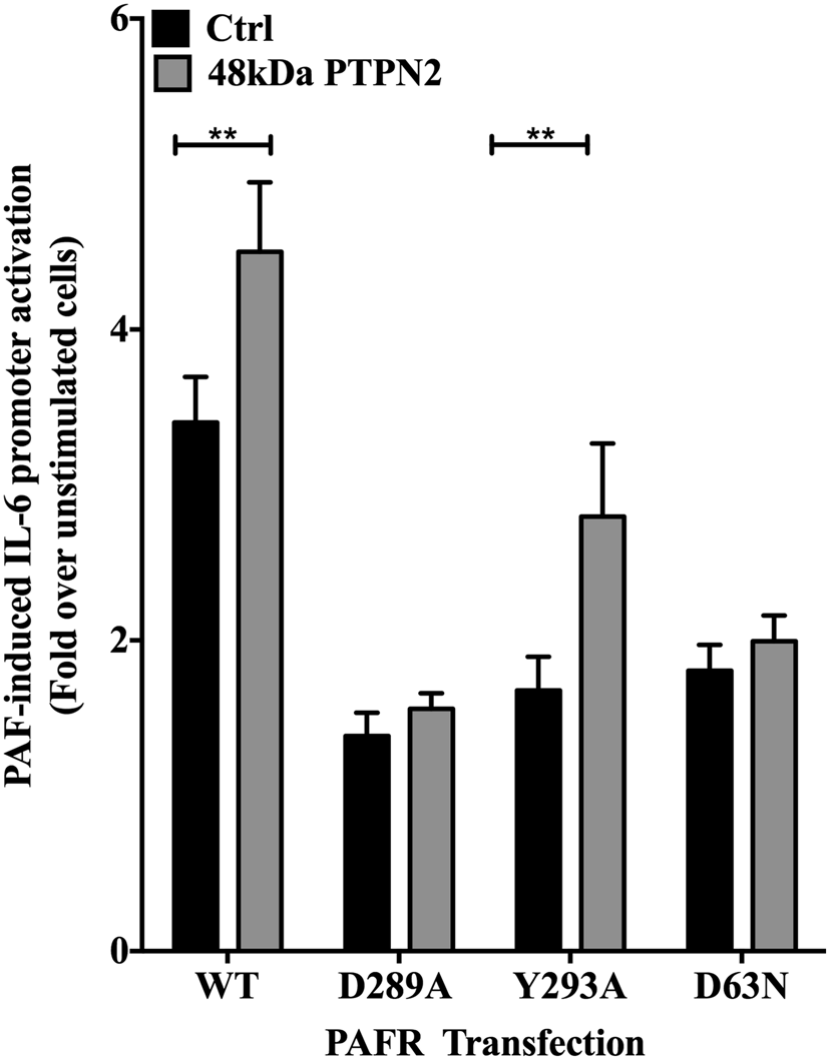
48 kDa PTPN2 modulation of PAF-induced hIL-6 promoter activity does not depend on G-protein activation. HEK-293 were transiently co-transfected with the hIL-6-luc, wild type or mutant PAFRs and control vector (pcDNA3) or the 48 kDa PTPN2 constructs. Cells were incubated overnight in DMEM-0.2% BSA, stimulated with PAF (100 nM) or vehicle for 6 h and luciferase activity was measured. The data presented are mean ± SEM of 4–5 independent experiments performed in triplicate. Significance was established with paired Student’s t test. **p < 0.01

We then examined whether G-protein signaling was necessary for PTPN2-mediated effects. We used HEK-293 cells co-transfected with PTPN2 and WT or mutant PAFR cDNAs. We have previously shown that the PAFR substitution mutants D289A, Y293A and D63 N are uncoupled from G proteins [69]. Consistent with the experiments shown above, a significantly higher activation of the hIL-6 promoter was observed in WT PAFR-PTPN2 co-transfected cells, than in those transfected only with WT PAFR (Fig. 4). PAF stimulation of all three G-protein-uncoupled mutants induced significantly less hIL-6 promoter activation than the WT, indicating the importance of G-proteins in IL-6 induction. PTPN2 co-transfection with D63N or D289A mutant receptors did not affect the levels of IL-6 transcriptional activation after PAF stimulation; however, IL-6 transcription was significantly augmented by PTPN2 when co-transfected with the Y293A PAFR mutant. These data indicate that G-protein signal transduction is not the direct target of PTPN2. These results also support those obtained with the Jak2 inhibitor, AG490, given that PAF-stimulated Jak2 activation is independent of G-proteins [18]. Taken together, these results suggest a role for the 48 kDa isoform of PTPN2 upstream of all tested pathways and that could, at least in part, be mediated through G-protein-independent pathways. We are aware that our interpretations must be made with caution due to the possible limited specificity and efficacy of the pharmacological approach used here. However, the fact that none of the inhibitors tested induced a significant variation in luciferase activity in cells stimulated with TNFα (Additional file 1: Fig. S4), indicates a certain selectivity of the inhibitors, given that the activation of the IL-6 promoter by TNFα is mostly dependent on NF-kB, which is activated independently of these pathways [70, 71].

### Modulation of PI3K pathway by PTPN2

Among pathways known to be activated early in PAFR signaling, the PI3K/Akt pathway is an interesting candidate to investigate for 48 kDa PTPN2 involvement, as it is downstream of G-proteins, arrestins and Jaks [72–76]. Studies have shown that PAF-induced PI3K activation is observed as early as 5 s post-stimulation [77] and that this kinase could be upstream of Src [78], Erk [79] and NFκB activation [79, 80] in PAF-stimulated cells. These three pathways are all important activators of the IL-6 promoter [81]. Moreover, an G-protein-independent but arrestin-dependent activation of the PI3K/Akt pathway could provide an explanation to our results showing that PTPN2 increased PAF-induced IL-6 promoter activation only for the G-protein uncoupled PAFR mutant Y293A: this mutant is still able to recruit the Arrestin-2 whereas the Y289A mutant, which has an impaired internalization, could not [67, 69]. The D63N mutant also has decreased internalization levels which may be due to an impaired recruitment of arrestins since over expression of arrestins rescued the internalization of the receptor [82].

**Fig. 5:**
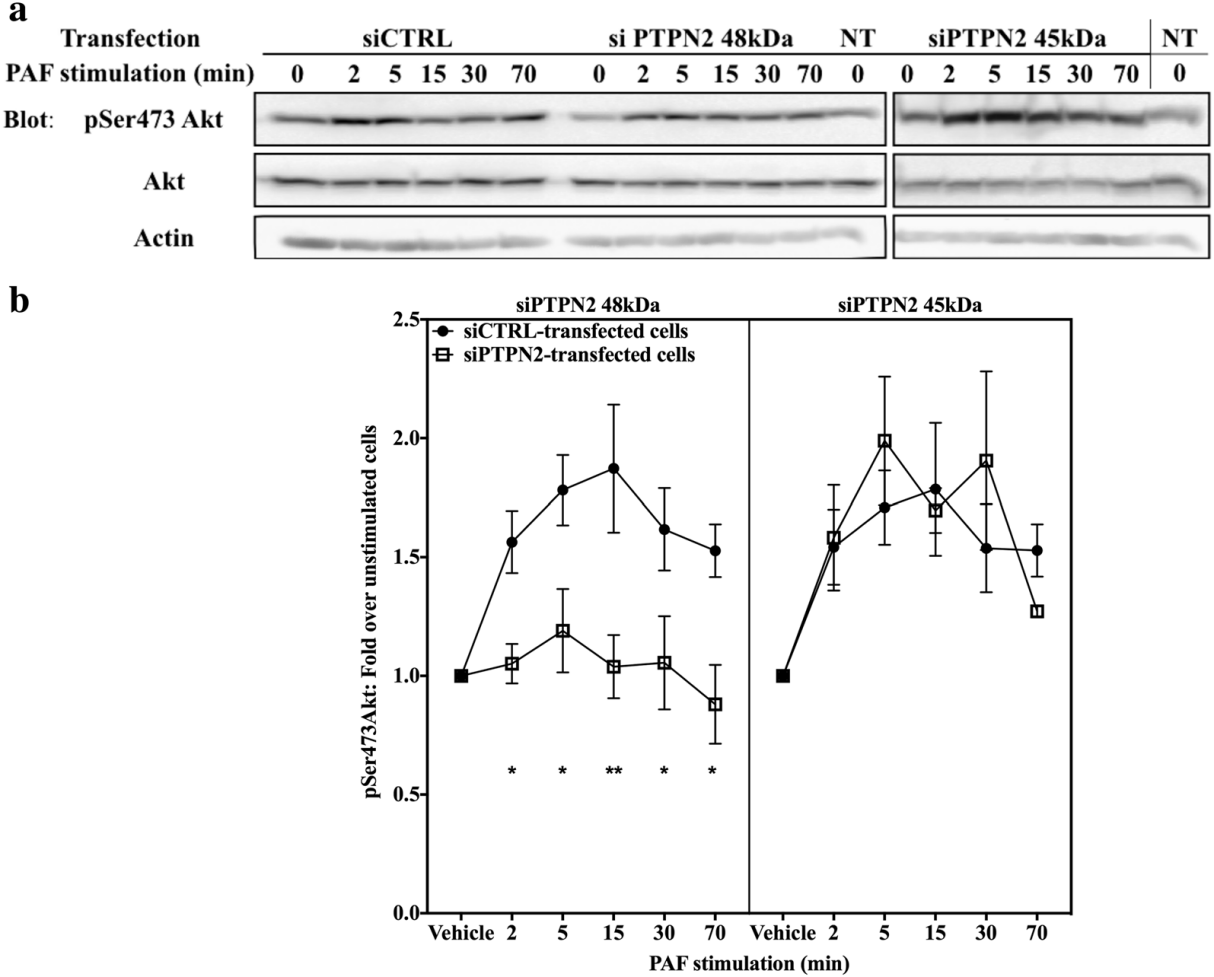
48 kDa PTPN2 modulates PAF-induced Akt activation in iMoDCs. Immature monocyte-derived dendritic cells (iMo-DCs) were transfected with control siRNAs (siCTRL) or against isoforms of PTPN2 (siPTPN2 48 kDa or 45 kDa) on day 4 and 5 before being collected on day 7 for experiments. iMo-DCs were starved for 5 h in RPM1 + 0.2% BSA and stimulated with PAF (1 nM) for indicated times. Whole cell lysates were separated by SDS-PAGE, transferred to nitrocellulose membranes and blotted overnight with anti-pSer473Akt, anti-Akt and anti-actin antibodies. **a** Representative blots and **b** compilations of at least 4 independent experiments are shown. The data presented are a mean ± SEM. Significance was established with two-way ANOVA with Sidak post-test. *p < 0.05, **p < 0.01

We, therefore tested whether the PI3K pathway was involved in PAF-induced IL-6 promoter activation. In HEK-PAFR the PI3K inhibition by both pharmacological inhibitors and the transfection of the p85∆SH2 (Additional file 1: Fig. S5A) strongly decreased PAF-induced IL-6 transactivation, indicating that in HEK-PAFR which express, even if weakly, the p110α, β and δ isoforms but not the 110γ isoform of PI3K [83, 84], PAF activated the hIL-6 promoter via a class IA PI3K-dependent pathway. We showed that PTPN2-mediated increase was dependent on Jak2 activity (Fig. 3) whereas G-protein activation was not absolutely necessary for the 48 kDa PTPN2-mediated IL-6 promoter increase (Fig. 4). We therefore examined whether PTPN2 could modulate the PI3K/Akt pathway by measuring the phosphorylation levels of its downstream effector, Akt, on serine 473 (pSer473) which is associated with a fully activated form of Akt [85]. In siCTRL- and siRNA PTPN2 45 kDa-transfected iMo-DCs, PAF-induced increase in pSer473 Akt levels was visible as soon as 2 min post-stimulation and was maintained for 30 min. pSer473Akt levels in PAF-stimulated cells were significantly lower in siPTPN2 48 kDa-transfected iMo-DCs compared to those in siCTRL-transfected Mo-DCs or siPTPN2 45 kDa-transfected cells (Fig. 5), and this, without significantly affecting pSer473 Akt basal levels (Fig. 5a and Additional file 1: Fig. S6A), suggesting that the PI3K pathway could be modulated by PTPN2 in a PAF- and isoform-specific manner. Consistent with these results, a significant modulation of pSer473 Akt was observed only when HEK-PAFR were transfected with the 48 kDa isoform (Additional file 1: Fig. S5C), and this, without significantly affecting pSer473 Akt basal levels (Additional file 1: Fig. S5B, C). This modulation of the PI3K/Akt could provide an explanation for the decreased SFK activation found in cell transfected with the D182A-48 kDa PTPN2 isoform (Additional file 1: Fig. S3), given that PI3K could be upstream of Src in PAF-induced proliferation of ovarian cancer cells [78]. Modulation of Src activity by the PI3K/Akt pathway has also been reported in H2O2 stimulated cells [86]. Moreover, increasing PI3K/Akt activity by PTPN2 could also explained the increased ERK activation given that, in PAF-stimulated cells, ERK is known to be a downstream effector of the PI3K/Akt pathway [79, 87]. We therefore decided to pursue the investigation of PTPN2-mediated modulations of PI3K/Akt pathway in PAF-stimulated cells.

### Involvement of PKD in PTPN2-mediated effects

Next, the mechanism by which PTPN2 regulated the PI3K pathway was investigated by focusing on processes involved in class IA PI3K regulation. The p85 subunit of PI3K has been shown to be phosphorylated on tyrosine residues, increasing the lipid kinase activity of PI3K [88, 89]. This regulatory mechanism is also activated by GPCRs, since ghrelin-stimulated HEK-293 cells, stably transfected with GHSR1a, a GPCR coupled to G_i/o_ and G_q/11_, similarly to PAFR, could induce tyrosine phosphorylation of p85-PI3K [76], as did PAF in the ASK.0 cell line [90]. Given that PTPN2 increased the activation of PI3K/Akt pathway, a direct dephosphorylation of p85-PI3K does not fit our data. The same reasoning applies to many of the known PTPN2 substrates involved in PI3K activation, such as EGFR, leading to their exclusion as potential targets in the context of PAFR stimulation [91]. Src can activate the p85-PI3K pathway [88, 89] and can be negatively regulated by PTPN2 [29, 66]. However, 48 kDa PTPN2 seemed to increase Src activation as seen by Western blots and luciferase assays, excluding this kinase as a primary substrate for PTPN2.

We therefore explored alternate substrates for PTPN2. PKD seemed a potential target since it has been reported that activated PKD can inhibit PI3K activation by increasing the p85-PI3K subunit phosphorylation levels on either the Ser154, thus increasing its association with PTEN and the lipid phosphatase activity of the latter [92, 93], or on Ser652, impairing a conformational change necessary for PI3K activation induced by the binding of the p85 subunit to phospho-tyrosine peptides [94]. PKD is activated by sequential tyrosine phosphorylations by Src (Tyr95) [95] and by Abl (Tyr463 in the PH domain), suggesting a target site for PTPN2 [96]. Studies of the regulation of oxidative stress-induced pathways have demonstrated that PKD is activated by pervanadate, an irreversible inhibitor of PTPs [96] and that certain PTPs such as RPTPα can form a signaling complex with PKD and modulate indirectly its activity [97]. Furthermore, the human protein–protein interaction prediction database PIPs predicted an interaction between PKD and PTPN2 [98, 99].

**Fig. 6:**
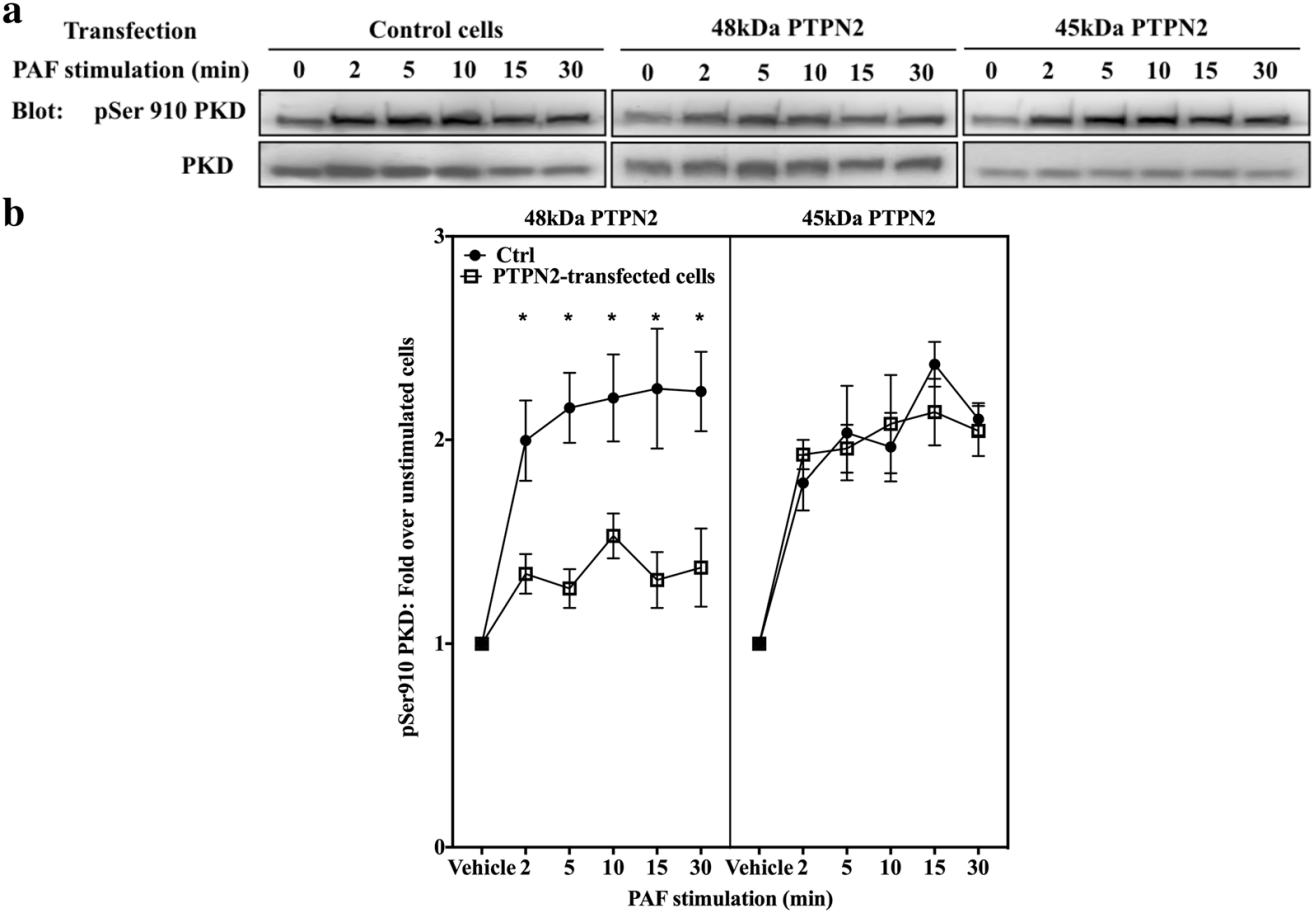
48 kDa PTPN2 modulates PAF-induced PKD activation in HEK-PAFR. **a** HEK-PAFR were transiently transfected with 48 kDa or 45 kDa PTPN2 cDNAs or control vector. Cells were incubated overnight in DMEM-0.2% BSA, then stimulated with PAF (100 nM) or vehicle for indicated times. Whole cell lysates were separated by SDS-PAGE, transferred to nitrocellulose membranes and blotted overnight with anti-pSer910 PKD then anti-PKD antibodies, after stripping. **a** Representative blots and **b** compilations of 3 independent experiments are shown. The data presented are a mean ± SEM. Significance was established with two-way ANOVA with Sidak post-test. *p < 0.05

We tested whether PTPN2 could modulate PAF-induced PKD activation. Western blots were performed with lysates obtained from HEK-PAFR transfected with a control, 45 kDa or 48 kDa WT PTPN2 vectors and blotted with anti-PKD antibodies or anti-pSer910 PKD antibodies, the phosphorylation levels at this site of auto-phosphorylation correlate with PKD kinase activity [100]. This site was chosen instead of the Ser744/748 site of phosphorylation in the activation loop, which is phosphorylated by PKC, since it has been shown that stimuli such as IFNα and angiotensin II can activate PKD by mechanisms that do not require the phosphorylation of these residues [101, 102]. PAF induced a strong and sustained phosphorylation of PKD in control and in 45 kDa PTPN2-transfected cells (Fig. 6). The phosphorylation on serine 910 was significantly decreased in 48 kDa PTPN2-transfected cells compared to both siCTRL- or siPTPN2 45 kDa-transfected cells (Fig. 6b).

**Fig. 7:**
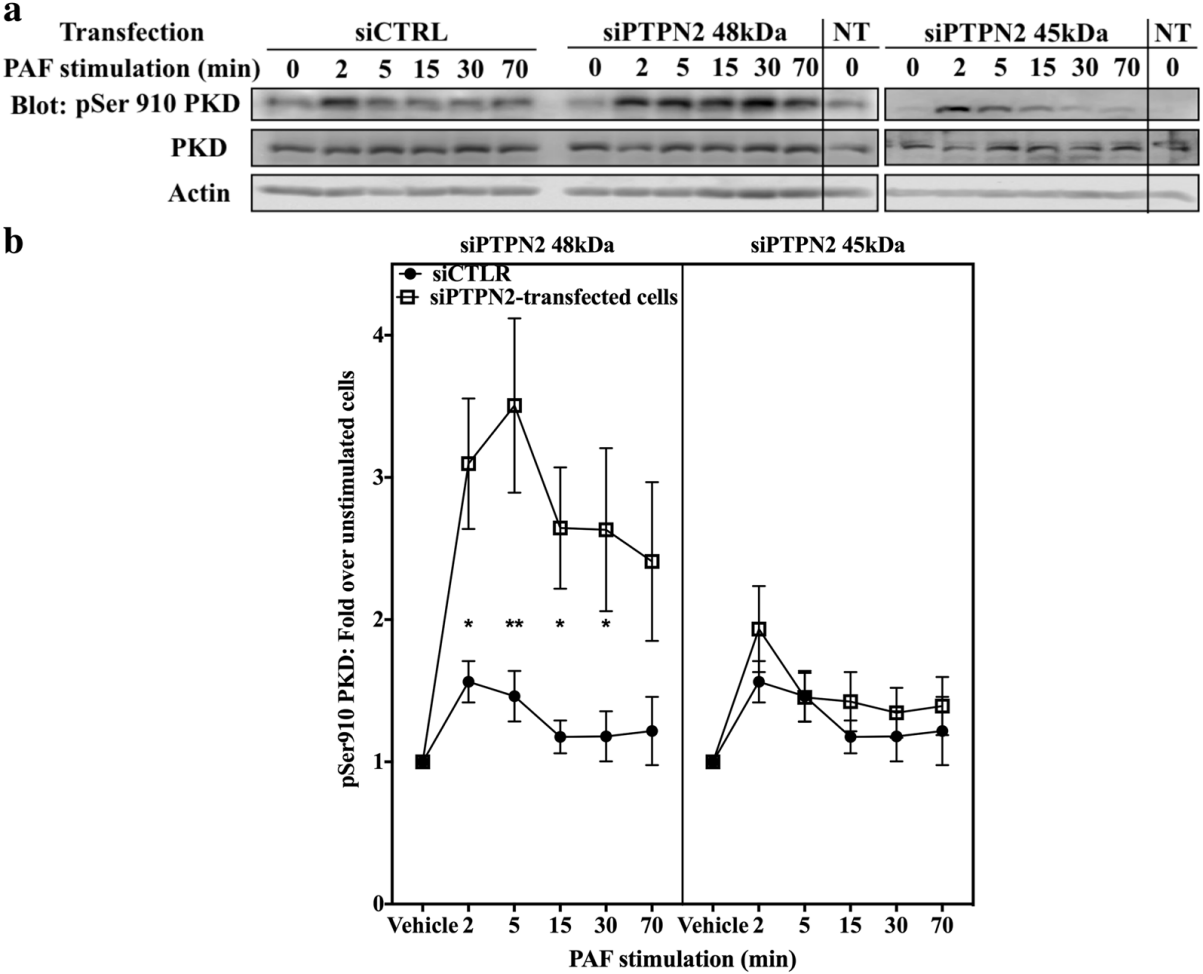
48 kDa PTPN2 modulates PAF-induced PKD activation in iMoDCs. Immature monocyte-derived dendritic cells (iMo-DCs) were transfected with control siRNAs (siCTRL) or siRNAs against isoforms of PTPN2 (siPTPN2 48 kDa or 45 kDa) on day 4 and 5 before being collected on day 7 for experiments; or not transfected (NT). iMo-DCs were starved for 5 h in RPM1 + 0.2% BSA and stimulated with PAF (1 nM) for indicated times. Whole cell lysates were separated by SDS-PAGE, transferred to nitrocellulose membranes and blotted overnight with anti-pSer910 PKD, anti-PKD and anti-actin antibodies. NT samples have been loaded on the same blots as PTPN2 samples, to serve as controls. **a** Representative blots and **b** compilations of at least 3 independent experiments are shown. Significance was established with two-way ANOVA with Sidak post-test. *p < 0.05, **p < 0.01

We then explored the modulation of PAF-induced PKD pathways by PTPN2 in iMo-DCs. siRNA-transfected iMo-DCs were stimulated with PAF for indicated times and Western blots were performed. PAF induced a transient phosphorylation of PKD on Ser910 in siCTRL and siPTPN2 45 kDa-transfected iMo-DCs (Fig. 7a, b). A significantly stronger phosphorylation of PKD was seen in siPTPN2 48 kDa-transfected cells than in siCTRL or siPTPN2 45 kDa-transfected iMo-DCs (Fig. 7a, b), and this, without significantly affecting basal pSer910 PKD levels (Fig. 7a, Additional file 1: S6B).

These results obtained in two different cell types led us to the conclusion that PAF could activate PKD and that PTPN2 could be involved, in an isoform-dependent manner, in the modulation of PKD activation. The differential localization of PTPN2 isoforms may contribute to the discrepancy between the 45 and 48 kDa isoforms in PKD modulation since PKD, a cytoplasmic protein in resting cells [103, 104], rapidly translocates to the plasma membrane after stimulation of GPCRs, such as bombesin [105] and fMLP [104] receptors. Both these locations would be consistent with 48 kDa, but not 45 kDa, PTPN2 locations observed in our experiments. On the other hand, the difference in phosphatase activity between both PTPN2 isoforms would not seem to contribute to the discrepancy in PKD modulation given that the 48 kDa isoform modulated PAF-induced pSer910 PKD levels within 2 min of stimulation. Altogether, our results suggest that PAF activated PKD and the presence of 48 kDa PTPN2 kept the activation at lower levels. This is the first time, to our knowledge, that PKD was shown to be activated by PAF; however, the mechanism by which PAF activated and PTPN2 decreased PKD phosphorylation is still unknown. It is possible that PTPN2 could decrease PKD activity either by direct dephosphorylation or by modulating SFK activation. However, according to our data, SFKs were not the primary targets of PTPN2 and therefore would act downstream of PTPN2 substrates. The exact mechanisms by which PAF activates Src or PKD are not clearly defined and therefore, by extension, the mechanisms by which PTPN2 could modulate them are also unknown and require further investigation.

**Fig. 8:**
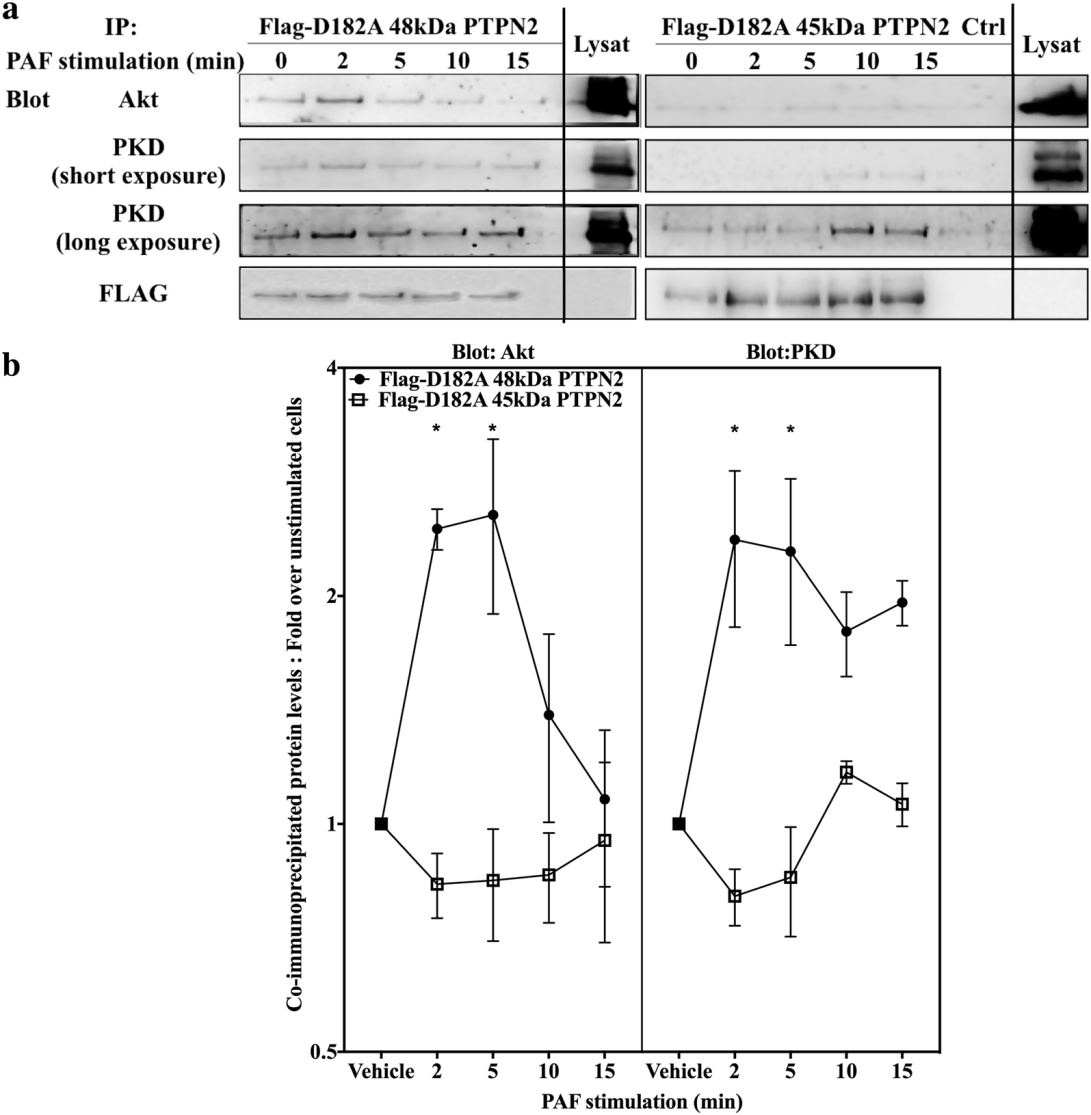
48 kDa PTPN2 forms a signaling complex with PKD and Akt. HEK-PAFR were transiently transfected with D182A-48 kDa or − 45 kDa PTPN2 constructs tagged with Flag or control vector (Ctrl). Cells were incubated overnight in DMEM-0.2% BSA, then stimulated with PAF (100 nM) or vehicle for indicated times. Stimulation was stopped on ice, cells were scraped and lysed. Whole cell lysates were submitted to immunoprecipitation using anti-FLAG antibodies coupled to agarose beads. **a** Precipitates were separated by SDS-PAGE, transferred to nitrocellulose membranes and blotted overnight with anti-Akt, anti-PKD or anti-Flag antibodies. Lysate samples have been loaded onto the same blots as immunoprecipitated PTPN2 samples and serve as loading controls. **a** Representative blots and **b** compilations of at least 3 independent experiments are shown. The data presented are mean ± SEM. Significance was established with two-way ANOVA with Sidak post-test. *p < 0.05

PAF-induced PKD regulation could be consistent with a model where the PI3K/Akt pathway would be under negative control of PKD and PKD would be regulated by a PTPN2-dependent pathway in PAF-stimulated cells. Therefore, a possible interaction between PTPN2, PKD and Akt was explored. Since a phosphatase-substrate interaction is very transitory, we used PTPN2 substrate-trapping mutants to reveal any pertinent interactions. HEK-PAFR were transfected with a control vector or Flag-tagged D182A 45 kDa or 48 kDa PTPN2 trapping mutants, and stimulated with PAF. Flag-tagged PTPN2 was immunoprecipitated with anti-Flag beads. Western blots were performed and membranes were blotted with anti-Akt or anti-PKD antibodies. Figure 8 shows that D182A 48 kDa PTPN2 co-immunoprecipitated with Akt and the amount of associated protein was modulated by PAF stimulation in a time-dependent manner. The maximal amount of Akt co-immunoprecipitated with D182A 48 kDa PTPN2 was found at 2 and 5 min post-stimulation, suggesting a transient interaction, which also indicates that the interaction would be independent of the phosphatase domain. In D182A 45 kDa PTPN2-transfected cells, PAF induced no significant variation in co-immunoprecipitated Akt levels. For PKD, D182A 48 kDa PTPN2 precipitated 2 times more of the endogenous protein at 2 and 5 min post-stimulation than what was found in unstimulated cells. PKD was still found in appreciable levels in the precipitate at 10 and 15 min post-stimulation. On the other hand, no significant modulation of PKD levels was observed with immunoprecipitated D182A 45 kDa PTPN2. This suggests that PAF increased the interaction between PTPN2 and PKD and, possibly, PTPN2 and Akt in an isoform-specific manner. This interaction was modulated in a time-dependent manner by PAF stimulation. Given that the data showing that D182A-PTPN2 mutants modulated IL-6 promoter transactivation (Additional file 1: Fig. S3E) are consistent with those obtained from experiments conducted with WT PTPN2 or siRNA, we conclude that the signaling complex found here with mutated PTPN2 could be physiologically relevant and that PAF induced the formation of signaling complexes containing PKD and Akt which could be modulated by PTPN2. This conclusion is consistent with data showing that Akt and PKD do have common partners. At this time, we have no data as to whether PTPN2 interaction with either protein could be direct. It has been shown that Akt can directly bind and phosphorylate 14-3-3ζ [106] which can also be bound by PKD [107]. Src, which is activated more in cells that are transfected with the 48 kDa PTPN2, is another common partner between Akt and PKD, since Src can phosphorylate and activate both proteins [108].

**Fig. 9:**
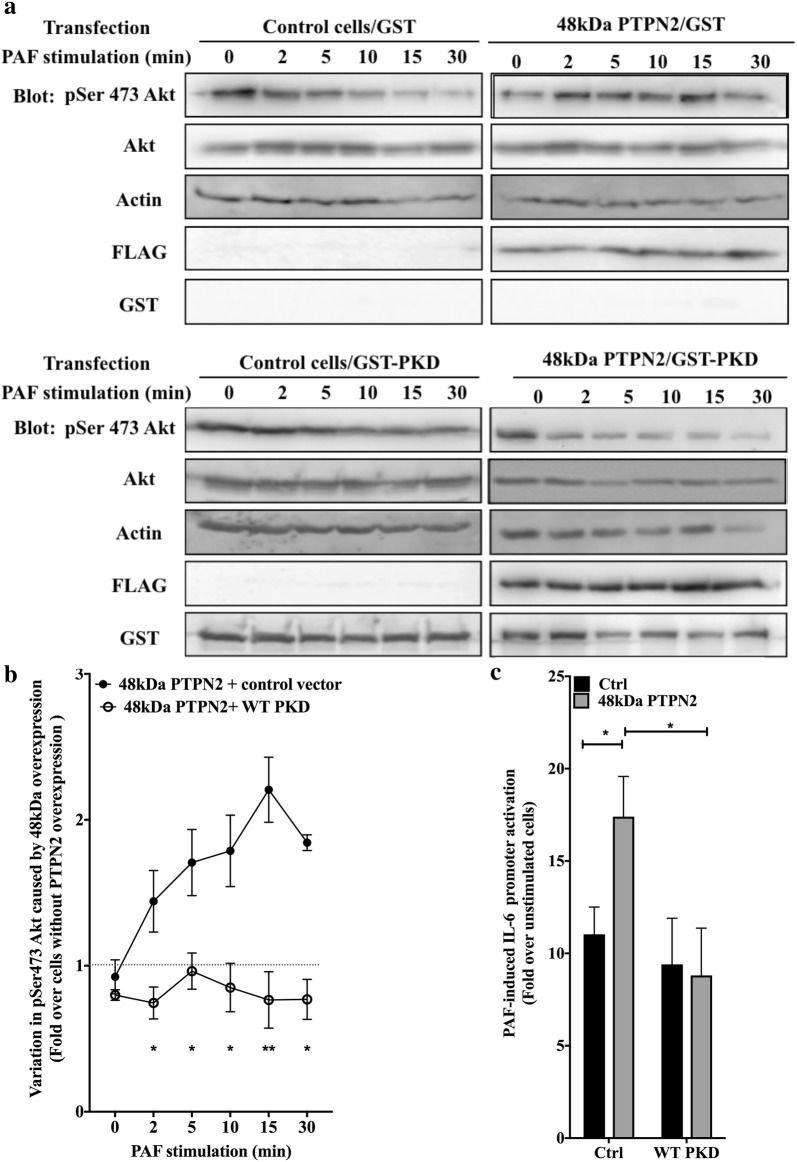
48 kDa PTPN2 modulates Akt activation via a PKD-dependent pathway. **a**, **b** HEK-PAFR were transiently transfected with 48 kDa or 45 kDa PTPN2 constructs tagged with FLAG, PKD tagged with GST or control vectors (pcDNA3 or pcDNA3-GST). Cells were incubated overnight in DMEM-0.2% BSA, then stimulated with PAF (100 nM) or vehicle for indicated times. Stimulation was stopped on ice, cells were scraped and lysed. Whole cell lysates were separated by SDS-PAGE, transferred to nitrocellulose membranes and blotted overnight with anti-pSer473Akt, anti-GST, anti-actin, anti-FLAG and anti-Akt antibodies. Results shown are a compilation of 3 independent experiments, expressed as variations of Akt phosphorylation due to PTPN2 overexpression where values of pAkt levels of control cells (without PTPN2) are set to 1 (Dotted line) for each indicated stimulation time. **c** HEK-PAFR were transiently co-transfected with the hIL-6-luc, control vector pcDNA3, 48 kDa PTPN2 or PKD constructs. Cells were incubated overnight in DMEM-0.2% BSA and stimulated with PAF (100 nM) or vehicle for 6 h and luciferase activity was measured. The data presented are mean ± SEM of at least 3 experiments performed in triplicate. **a**–**c** The data presented are mean ± SEM. Significance was established with two-way ANOVA with Sidak post-test. *p < 0.05

To verify whether the PTPN2-mediated up-regulation of PI3K/Akt pathway depended on PKD activity, Western blots were performed with lysates obtained from HEK-PAFR transfected with either the 48 kDa PTPN2, control vector, PKD or a combination of both proteins (Fig. 9a, b). The over-expression of PKD alone did not have a significant effect on pSer473Akt levels but it did abolish the 48 kDa PTPN2-induced modulation of PAF-induced pSer473Akt levels, suggesting that the altered phosphorylation levels could be due to a modulation in PKD activation seen in 48 kDa PTPN2-transfected cells.

To ensure that this modulation of PKD activation was relevant to PAF-induced hIL-6 promoter activation, luciferase assays were performed in HEK-PAFR transfected with a control vector, PTPN2 and WT PKD. The co-transfection of WT PKD was sufficient to significantly decreased PAF-induced IL-6 promoter activation in the 48 kDa PTPN2-transfected cells (Fig. 9c), suggesting this kinase could be involved in a negative feedback loop. Altogether, results presented here indicate that PTPN2 could modulate the PAF-stimulated PI3K/Akt pathway and hIL-6 promoter activation via the down-regulation of PKD activation.

We suggest that the mechanisms by which 48 kDa PTPN2 increased PAF-induced IL-6 expression in HEK-PAFR and iMo-DCs may be the same given that in iMo-DCs, a relatively small decrease in the expression of the 48 kDa isoform of PTPN2 due to siRNA transfection was sufficient to increase PAF-induced phosphorylation of PKD, to decrease the pSer473Akt levels and PAF-induced IL-6 mRNA levels. In return, the results obtained in iMo-DCs also suggest that those obtained in HEK-PAFR were not an artefact caused by overexpressed proteins, reinforcing the hypothesis that each isoform of PTPN2 modulates specific cell signalling pathways. This is consistent with reports in the literature, for example, that the dominant negative form of the 48 kDa isoform, but not the 45 kDa one, could inhibit anchorage-independent cell growth of transformed fibroblasts [34]. In contrast, the 45 kDa isoform is involved in nuclear de-phosphorylation of STAT6, the nuclear localization of phosphorylated STAT6 excluding a role for the 48 kDa isoform in this process [33]. In the human cell line HeLa, the 45 kDa isoform is involved in the modulation of Akt and SFK activation, after a stimulation with EGF and in the negative regulation of Jak1 activity after adhesion to collagen, whereas the 48 kDa isoform is not involved in the modulation of these pathways [109]. Thereby, our results may contribute to the elucidation of the differential involvement of these isoforms in receptor-induced signalling. In addition, our data are also among the few reports showing a predominant role for the 48 kDa isoform over the 45 kDa isoform in receptor signaling.

## Conclusion

**Fig. 10:**
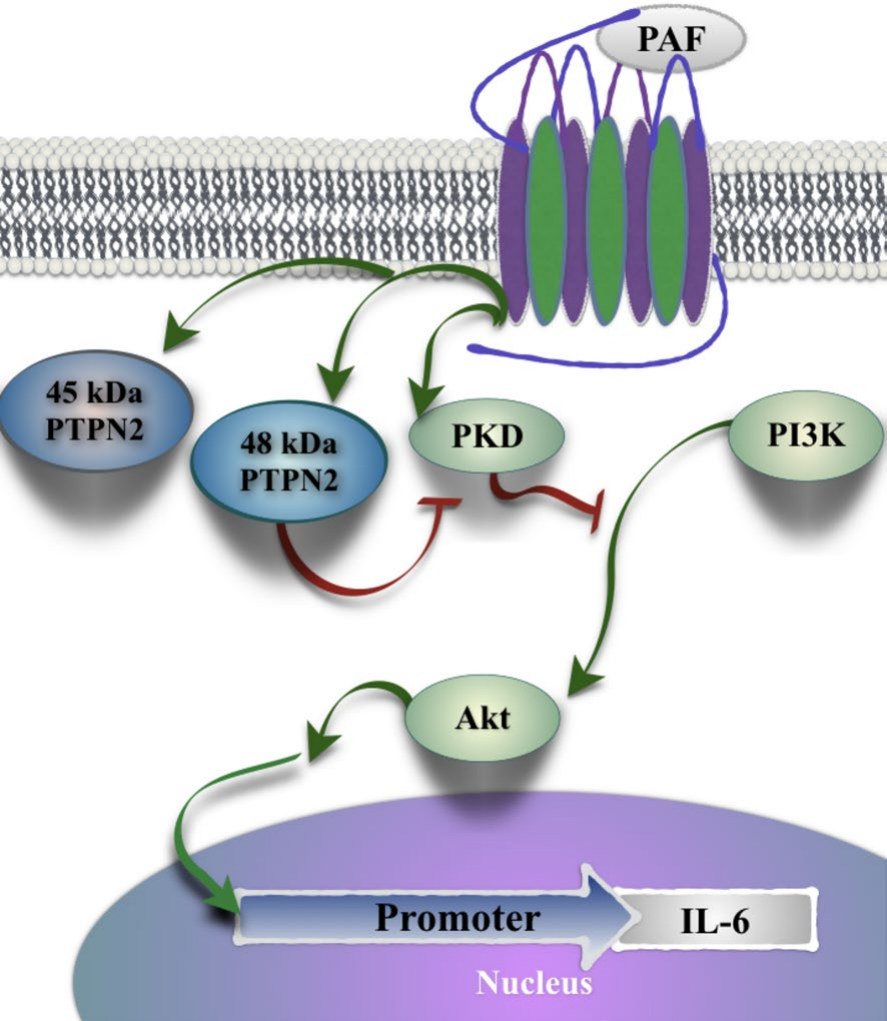
Schematic representation of pathways involved in PAF-induced PTPN2 modulation of IL-6 expression

Our results, presented in this report, highlight the fine regulation of PAFR signalling leading to IL-6 promoter activation as illustrated in Fig. 10. PAF increased enzymatic activity of both PTPN2 isoforms but only the 48 kDa PTPN2 upregulated PAF-induced IL-6 promoter transactivation, and this increase was mediated by a PKD/PI3K/Akt pathway, where PKD could be the primary target of PTPN2. PKD de-phosphorylation would relieve a negative pressure on PI3K/AKT, increasing their activation and downstream regulation of IL-6 transcription. Data obtained suggest that the differential localization of the PTPN2 isoforms may, in part, explain the isoform-specific modulation of PKD activation levels. Pathways leading to hIL-6 promoter activation, regulated by the 48 kDa isoform of PTPN2, seem to be specifically triggered by PAFR stimulation in our systems, as this isoform did not modulate TNFα-stimulated hIL-6 promoter activity. This novel observation of regulation of PAFR-mediated signaling could possibly be an interesting pathway to be targeted by therapeutic approaches in atherosclerosis, where PAF seems to act at every stage of the disease. In fact, by targeting PTPN2, we can expect to modulate signal intensity, leading to modified gene expression, at least for IL-6. Further research will be needed to determine whether effects observed here could be extended to other PAFR-mediated processes like migration, adhesion or gene activation, indicating an overall increase in PAFR-mediated cell activation, or whether this effect is restricted to specific pathways.

## Materials and methods

### Reagents and chemical products

PAF (C-16) was from Cayman Chemicals (Ann Arbor, MI) and TNF-α was from PeproTech (Ottawa, ON Canada). Concanavalin A, U-0126, Ag490, GF109230X, PP2, SP600125 (c-Jun kinase [JNK] inhibitor II), and wortmanin were from Calbiochem (San Diego, CA USA). LY 294002 was purchased from Biomol (distributed by Cedarlane, Canada). cOmplete, Mini EDTA Free Protease Inhibitor Tablets BSA and ATP were from Roche Diagnostics (Laval, QC Canada). DMEM, 4′,6-diamidino-2-phenylindol dilactate (DAPI) and d-Luciferin Na^+^ salt were from Invitrogen (Burlington, ON Canada). Anti-vinculin (mouse, RRID: AB_477617), anti-FLAG gel affinity (RRID: AB_10063035) and anti-Flag (RRID: AB_259529) antibodies (Abs), Paraformaldehyde (PFA) and BSA (essentially IgG-free for Western blotting) were from Sigma-Aldrich (Oakville, QC Canada) and TransIT LT1 from Mirrus (from Medicorp, Montréal, QC Canada). Fetal bovine serum (FBS) was from PAA Laboratories (Etobicoke, ON, Canada). Amersham ECL Select Western Blotting Detection Reagent and nitrocellulose membranes (Hybond ECL) were from GE (GE Heathcare, Piscataway, NJ USA). The MILLIPORE Re-Blot Plus Western Blot Strong Antibody Stripping Solution was from Fisher (Ottawa, ON Canada). All enzymes used were from New England Biolabs, Ltd. (Pickering, ON Canada). Anti-HA (Y-11) (polyclonal rabbit antiserum (RRID: AB_631618), Anti-Actin I-19 (RRID: AB_630836), anti-phospho-Erk (RRID: AB_627545), anti-Erk (RRID: AB_631453), mouse anti-PTP1B RRID: AB_2174955 and anti-GST (RRID: AB_627677) Abs were from Santa Cruz (La Jolla, CA, USA). Anti-pSer910 PKD (RRID: AB_330841), -PKD (RRID: AB_2268946), -Akt (RRID: AB_329827) and -pSerS473-Akt (RRID: AB_329825) Abs were from Cell Signaling (New England Biolabs Ltd., Pickering, ON Canada). Mouse anti-TC-PTP Ab (RRID: AB_2289092) was from Millipore (Mississauga, ON Canada). Vectashield Mounting Media was from Vector Laboratories (Burlington, ON, Canada) and p-nitrophenyl phosphate (pNPP) was from ProteoChem (Loves Park, IL, USA).

### siRNA sequences

We used a mix of 3 siRNA duplexes that we designed using siRNA Wizard Guidelines (http://www.sirnawizard.com) and Ambion website. For PTPN2, transcript 1, first siRNA is against the mRNA region 1464–1484 (5′-GCUUCUGCACUAGUAACUGAC-3′), the second, against region 2215–2235 (5AAUUCUCCUUACUGGGAUAGU-3′) and the last one, against the region 1724–1744 (5′-AUUUCACCUGACCAACAUUGA-3′). For PTPN2, transcript 2, first siRNA is against the mRNA region 1427–1445 (5′-GCCCACUCCGGAAACUAAAUU-3′), the second, against region 1342–1362 (5′GAUUGACAGACACCUAAUAUU-3′) and the last one, against the region 1617–1636 (5′AUUAGGAGAGAUUACUUUGUU-3′). SiRNA duplexes were from Sigma-Aldrich, with a UU overhang. SiRNA controls (mission siRNA Universal Negative control #1) were from Sigma-Aldrich (Oakville, ON Canada).

### Plasmid constructs

#### 45 kDa and 48 kDa PTPN2 constructs

First, human 45 kDa and 48 kDa PTPN2 were inserted into the pCDNA3 vector. For this, human 48 kDa PTPN2 from Open Biosystems (IHS1382-8391079, Incyte Easy-to-Spot Human cDNA Clone Id: LIFESEQ 3492721) was amplified by PCR using 5′primer (5′-GTT GCT AGC GCC ACC ATG CCC ACC ACC ATC GAG CGG-3′) and a 3′primer (5′-CAA GGT ACC TTA TAG GGC ATT TTG CTG AAA-3′) which sequences correspond respectively to the first seven amino acids of both PTPN2 isoforms and the last amino acids specific to the 48 kDa PTPN2 isoform. The 5′ primers contain a Kozak sequence and a site for NheI digestion. 3′ primers contain a site for KpnI. PCR products were inserted into the pcDNA3 poly-cloning site after KpnI/NheI vector digestion. Integrity was confirmed by sequencing (Centre d’innovation Génome Québec et Université McGill, Montreal, QC Canada). The same strategy was applied for the generation of the 45 kDa PTPN2-coding vector. DNA was from Open Biosystems (EHS1001-4877 cDNA: IMAGE Human cDNA clone Clone Id: 3872164) using the 5′primer (5′-GTT GCT AGC GCC ACC ATG CCC ACC ACC ATC GAG CGG-3′) and 3′primer (5′-CAA GGT ACC TTA GGT GTC TGT CAA TCT TGG-3′).

The 48 kDa and 45 kDa PTPN2 cDNAs were subcloned into a vector containing a FLAG-tag and a Kozak sequence previously digested with *Xba*I/*Age*I. Insert and vector were ligated and product identity was confirmed by sequencing.

#### GFP2 and venus PTPN2 constructs

48 kDa PTPN2 was amplified using T7 primer (5′-TAA TAC GAC TCA CTA TAG GG-3′) and the 3′ primer (5′-TAG GTA CCG CTA GGG CAT TTT GCT GAA AA AAC AG-3′) which corresponds to the last eight amino acids of PTPN2 and excludes the stop codon. The PCR product and pGFP2-N3(h) linker (a gift of Dr. C. Le Gouill, Université de Montréal) was digested with NheI and KpnI. Insert and vector were ligated and identity of the ligation product was confirmed by sequencing. The same strategy was applied for the generation of the pGFP2 45 kDa PTPN2-coding vector and GFP2- tagged D182A 45 and 48 kDa PTPN2.

For venus-tagged D182A PTPN2 constructs, pGFP2-D182A 45 and 48 kDa PTPN2 constructs and pCDNA3.1 Zeo(+)-Venus were digested with *Age*I and *Not*I-HF. Inserts and vectors were ligated and identity of the ligation product was confirmed by sequencing.

#### D182A PTPN2 constructs

Substrate trapping mutants D182A 45 and 48 kDa PTPN2 were generated by PCR amplification of the pCDNA3-kozak_hPTPN2 constructs using the primer extension method. In the first round, the pcDNA3 48 kDa PTPN2 construct described above was amplified in the common region of both isoforms using NEB Phusion DNA Polymerase (New England Biolabs Ltd., Pickering, ON Canada), the 5′ primer (5′-TAT GCT AGC GCC ACC ATG CCC ACC-3′) and the 3′ primer (5′-TGG TGA TTC AGG GAC TCC AAA GGC TGG CCA GGT AGT ATA ATG AAA-3′ or the 5′ primer (5′-TTT CAT TAT ACT ACC TGG CCA GCC TTT GGA GTC CCT GAA TCA CCA-3′) and the 3′ primer (5′-CTC TCA CTG TTC TCC TCC ATT G-3′). In the second round, mutant fragments were generated using PCR products, the 5′ primer (5′-TAT GCT AGC GCC ACC ATG CCC ACC-3′) and the 3′ primer (5′-CTC TCA CTG TTC TCC TCC ATT G-3′). PCR fragments and pCDNA Flag tagged-5 or 48 kDa PTPN2 or venus-tagged 45 or − 48 kDa PTPN2 were digested with *Nhe*I and *Bst*EII. Inserts and vectors were ligated and identity of the ligation product was confirmed by sequencing.

P85Δ-PI3K plasmid was a gift from Dr. Patrick McDonald (Université de Sherbrooke) and is described elsewhere [110]. PKD plasmids were kindly provided by Dr. Fernand-Pierre Gendron (Université de Sherbrooke) and were described elsewhere [111].

### Cell culture

HEK-293 (CRL. 1573, American Type Culture Collection (ATCC), Rockville, USA) stably transfected with pIRES_puro_PAFR_HA (HEK-PAFR) were grown in DMEM, 5% FBS at 37 °C 5%CO_2_ in a humidified atmosphere with puromycin, 5 µg/ml, penicillin, 60 µg/ml and streptomycin, 100 µg/ml.

iMo-DCs were generated from monocytes obtained from healthy volunteers after informed, written consent in accordance with a Université de Sherbrooke Human Ethics Review Board-approved protocol (#93-04), adhering to the Helsinki agreement. Monocytes were isolated from peripheral venous blood after enrichment by dextran sedimentation followed by purification by density gradient centrifugation on Ficoll-Hypaque and adhesion on autologous serum-coated Petri dishes. Following intensive washings, adherent cells were recovered and cultured for 7 days in RPMI 1640 supplemented with 10% FBS, rhGM-CSF (20 ng/ml) and rhIL-4 (20 ng/ml). Medium was changed after 24–48 h by removing half of the volume and adding the same volume of new medium. On day 5, the medium was changed by removing 1.5 ml and replacing with medium containing only 10 ng/ml of each cytokine. Finally, at the end of day 6, 3 ml of RPMI 5% FBS without any cytokines was added to begin the starvation of the cells. On day 7, cells were collected and stimulated as described below. Where mentioned, cells were transfected with siRNAs: on day 4, a solution of 2 µM siRNA in RPMI 1640 (75 µl) containing 2.3 µl TransiT LT was added to 4.8 × 10^6^ cells (1 well of a 6 -well plate, 1.5 ml, final 100 nM siRNA) and incubated for 6 h at 37 °C, 5% CO_2_. After that, 1.5 ml of fresh medium with 20 ng/ml of each cytokine and 10% FBS was added. A second transfection of siRNAs was done 30 h after the first one: 1.53 µM siRNA in RPMI 1640 (75 µl) containing 2.3 µl TransiT LT was added to 4.8 × 10^6^ cells (1 well of a 6-well plate, 1.5 ml, final 75 nM siRNAs) and incubated for 6 h at 37 °C, 5% CO_2_. After that, 1.5 ml of fresh medium with 10 ng/ml of each cytokine and 10%FBS was added.

### Transient transfection and luciferase assay

HEK-PAFR were plated 12 h before transfection in 24-well plates. Cells were transiently transfected with 200 ng of wild type (WT) or mutant forms of pcDNA3–45 kDa or − 48 kDa hPTPN2 and 40 ng of pGL3-hIL-6-promoter (hIL-6-luc) per well using 0.75 µl of TransIT LT1 transfection reagent according to the manufacturer’s instructions. After 24 h in DMEM 5% FBS without puromycin, medium was changed and cells were incubated in DMEM 0.2% BSA for 16 h. The cells were lysed 6 h after stimulation by PAF at indicated concentrations. Where mentioned, cells were pre-treated for 20 min with inhibitors at indicated concentrations. Luciferase activity in lysates was measured as described before [112], using a Sirius luminometer (Berthold detector systems, Montreal, QC, Canada).

### Western blot analysis

HEK-PAFR were plated 12 h before transfection in 6-well plates. Where mentioned, cells were transiently transfected with 625 ng of pcDNA3-human WT or mutant D182A PTPN2 and 167 ng the PKD construct or vector, per well, using 3 µl of TransIT LT1 transfection reagent according to the manufacturer’s instructions. After 24 h in DMEM 5% FBS without puromycin, medium was changed and cells were left in DMEM 0.2% BSA for 16 h. Cells were then stimulated with 100 nM PAF for indicated times and the reaction was stopped on ice with ice-cold PBS containing 4 µM Na_3_VO_4_ and 20 mM NaF. Cells were scraped, centrifuged and lysed by adding ice-cold lysis buffer containing inhibitors (50 mM Tris HCl, pH 7.4, 150 mM NaCl, 1 mM EDTA, 1% TRITON X-100, 2 mM NaF, 4 mM Na_3_VO_4_, 10 nM calyculin A and cOmplete, Mini Protease Inhibitor Tablet) for 20 min on ice before being frozen at − 80 °C.

When the samples were thawed, they were centrifuged, 4× loading buffer (40% glycerol, 258 mM Tris–HCl pH 6.8, 8% SDS, 0,008% bromophenol blue, 20% 2-mercaptoetanol) was added to the supernatants, proteins were separated on 10% SDS-PAGE and subsequently transferred to a nitrocellulose membrane (Hybond, GE). Membranes were blocked with 5% milk in Tris-buffered saline (TBS) with 0.1%Tween 20 (TBS-T) before overnight blotting at 4 °C in TBS-T 5% milk with appropriate antibodies. For phospho-proteins, membranes were washed 3 × 5 min in TBS-T after blocking in milk, and blocked again for 20 min in TBS-T 5% BSA before overnight blotting at 4 °C in BSA. Finally, HRP-coupled secondary antibody (Cell Signalling) was added for 1 h, protein expression was revealed by ECL chemiluminescence detected with Versadoc (BioRad). Signal intensity was analyzed with ImageJ 1.43× software (National Institutes of Health, Bethesda, MD, USA). Data obtained were normalized and shown in fold increases over unstimulated as calculated using:

$$Fold\;increase\text{:}\frac{{normalized\;phospho - protein\;levels_{stimulated} }}{{normalized\;phospho - protein\;levels_{unstimulated} }}$$

where normalized phospho-protein levels were calculated as

$$normalized\;phospho-protein\;levels \text{=}\frac{{phospho - protein\;levels }}{{total\;protein\;levels }}$$

### RNA isolation and real-time quantitative PCR

RNA was obtained using Trizol reagent (Invitrogen, Burlington, ON, Canada) according to the manufacturer’s instructions. After quantification, 1.0 μg of RNA was converted to cDNA, QuantiTect Reverse Transcription Kit, according to the manufacturer’s instructions (Qiagen, Mississauga, ON, Canada). GAPDH, IL-6 and PTPN1 expression was measured using real-time PCR performed on a Rotor-Gene 3000 (Corbett Research, Kirkland, QC, Canada) as described previously [113]. The following oligonucleotide primer sets were obtained from IDT (Coralville, IN, USA): human RPL13A; forward 5′-GTG CGT CTG AAG CCT ACA AG-3′/reverse 5′-TCT TCT CCA CGT TCT TCT CG-3′; human GAPDH: forward, 5′-GAT GAC ATC AAG AAG GTG GTG AA-3′/reverse, 5′-GTC TTA CTC CTT GGA GGC CAT GT-3′; human IL-6: forward, 5′-GTG TGA AAG CAG CAA AGA GGC-3′/reverse, 5′-CTG GAG GTA CTC TAG GTA TAC-3′; human 48 kDa PTPN2: forward, 5′-ACT CTT GAC TGC AGG TTC-3′/reverse, 5′-TCA AGT GC AG GTT AA AAT CC-3′, for human 45 kDa PTPN2 5′-AAA GGC CAA GAT TGA CAG ACA-3′/reverse: 5′-AAG TCT TCT GCT GGT GGG TG-3′. Gene expression was normalized with GAPDH and/or RPL13A mRNA content and differences were calculated with the delta–delta (ΔΔ)Ct method according to the following formula: (ΔΔCt = [(Ct G.O.I.Ctl − Ct HK.G.Ctl) − (Ct G.O.I.STIM. − Ct HK.G.STIM.)]. Comparison of the expression of each gene between its control and stimulated/siRNA transfected state was determined by ΔΔCt. Results were then transformed into fold variation measurements: fold increase − 2ΔΔCt.

#### Microscopy

HEK-PAFR were grown overnight on poly-l-lysine-coated coverslips in DMEM, 5%FBS, transfected with 400 ng Venus-tagged pCDNA3-D182A-hPTPN2 and 1.5 µl Transit LT1. After 30 h, cells were starved for 7 h in DMEM 0.2% BSA and stimulated with 100 nM PAF for indicated times. Cells were fixed with 2.5% paraformaldehyde (PFA) for 15 min at room temperature (RT), then placed in 0.5% (v/v) Triton X-100 in PBS for 10 min RT. After washing, the coverslips were mounted on slides using Vectashield containing DAPI. Sequential acquisitions at 488 and 555 nM were performed with a Plan Apo 60× oil immersion objective NA 1.42 on an inverted spectral scanning confocal microscope FV1000 (Olympus, Tokyo, Japan). Images were acquired the same day, typically from 4 different optical fields for each experimental condition, using identical settings of the instrument. Images were analyzed with the JaCop Image J plug in.

### Colorimetric PTP assays

Colorimetric PTP assays were performed with a protocol adapted from McAvoy and Nairn [114]. Briefly, HEK-PAFR were transfected with Flag-tagged PTPN2 constructs and stimulated for indicated times. Cells were then lysed 20 min on ice with lysis buffer (50 mM Tris HCl, pH 7.4, 150 mM NaCl, 1 mM EDTA, 1% Triton X-100, 10 mM NaF, 1 mM Na_3_VO_4_, 10 µg/ml leupetine, 2 µg/ml pepstatin and cOmplete, Mini Protease Inhibitor Tablet) and PTPN2 was immediately immunoprecipitated from cleared lysates (at least 75 µg of lysate). Lysates were then incubated 2 h at 4 °C with 2 µg of mouse anti-Flag or mouse IgG control isotype Abs and 25 µl of a solution 50% protein-A Sepharose beads. Beads were recovered by centrifugation (3000 rpm, 1 min) and subsequently rinsed 2 times with ice-cold TBS-T. Samples were resuspended in 80 µl PTP buffer (25 mM HEPES, pH7.4, 1 mM EDTA, 100 µg/ml BSA, 0.01% Tween 20, 50 mM NaCl, 5 mM NaF, 2 mM DTT), distributed in 96-well plate (30 µl/wells) and incubated 5 min at RT. After that, 20 µl of pNPP solution was added (pNPP, 95 mg/ml in PTP buffer). Plates were incubated at 37 °C. Optical density (OD) was taken every 5–10 min at 405 nm (reference 800 nm) until the signal was saturated. OD was plotted as a function of time and slopes were taken in the linear portion of the curves. Hydrolysis rate was calculated with an extinction coefficient of 18,000 M–1 cm^−1^. After PTP experiments, reactions were loaded into SDS-PAGE, transferred to nitrocellulose membranes and blotted overnight with the mouse anti-TC-PTP Ab (RRID: AB_2289092) for normalization of the pNPP rate.

### Statistical analysis

Statistical significance was determined, using the Student’s t test for paired data (two-tailed) or two-way ANOVA with Sidak post hoc analysis, as appropriate, using PRISM7 software (GraphPad Software Inc, San Diego, CA). Differences were considered significant at p < 0.05.

## Additional file

**Additional file 1.** Additional figures.

## Data Availability

The datasets used and/or analyzed during the current study are available from the corresponding author on reasonable request.

## Abbreviations

PAF: platelet-activating factor
IL-6: interleukin 6
ER: endoplasmic reticulum
HEK-PAFR: HEK-293 cells, stably transfected with PAFR
Mo-DC: monocyte-derived dendritic cell
FAK: focal adhesion kinase
NLS: nuclear localization sequence
PKD: protein kinase D
pNPP: p-nitrophenyl phosphate
PTK: protein tyrosine kinase
PTPN2: protein tyrosine phosphatase non-receptor type 2
TNF-α: tumor necrosis factor alpha
TCPTP: T cell protein tyrosine phosphatase
WT: wild type
