# Supplementary material for: Regulation of platelet-activating factor-mediated interleukin-6 promoter activation by the 48 kDa but not the 45 kDa isoform of protein tyrosine phosphatase non-receptor type 2
Source: Cell Biosci. 2019 Jun 25;9:51. doi: 10.1186/s13578-019-0316-9 (PMC6593612; doi:10.1186/s13578-019-0316-9)
Supplement: Supplementary file 1 — Additional file 1. Additional figures. [file 13578_2019_316_MOESM1_ESM.pdf]

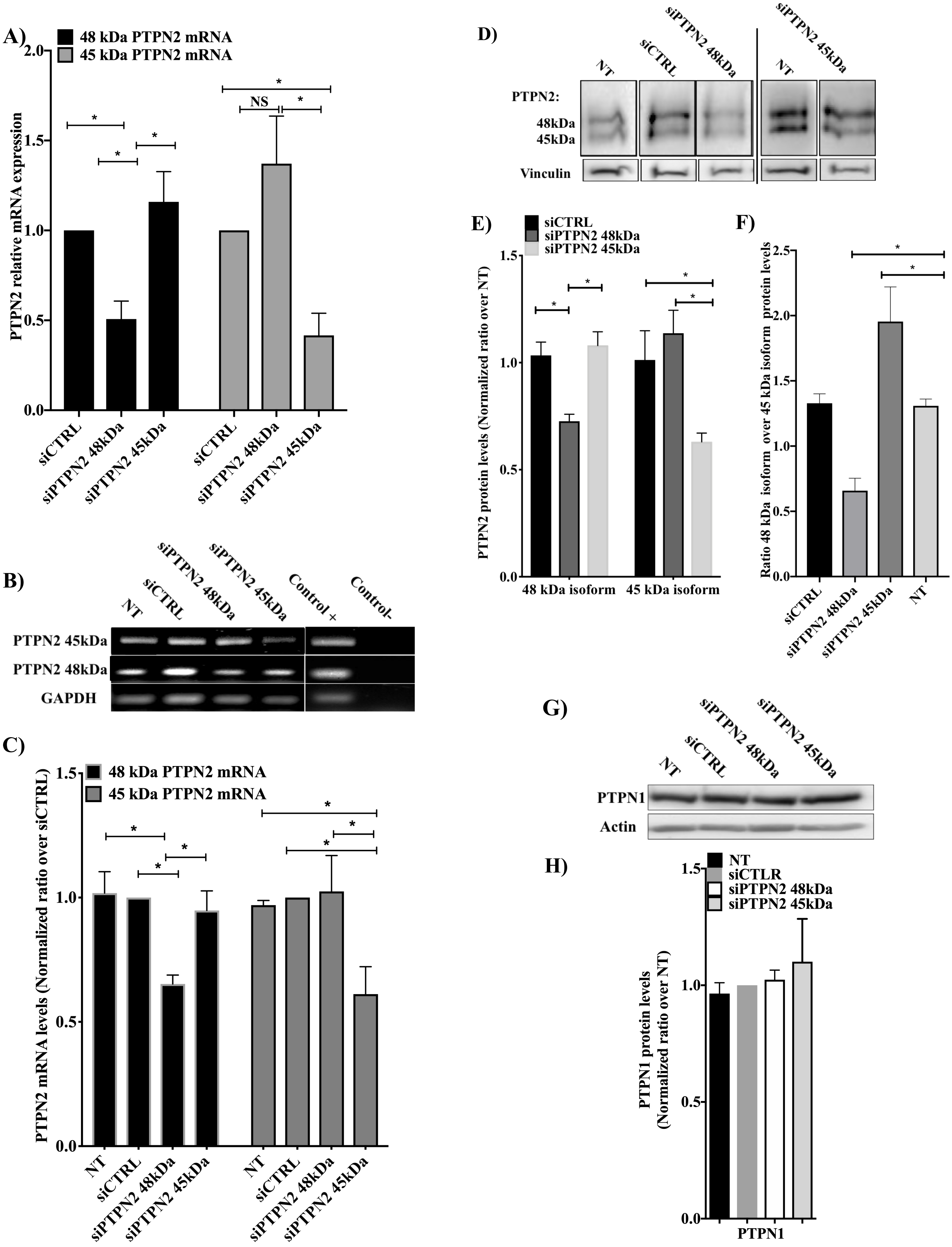
A)
48 kDa PTPN2 mRNA
45 kDa PTPN2 mRNA
PTPN2 relative mRNA expression
NS
siCTRL
siPTPN2 48kDa
siPTPN2 45kDa
B)
NT
siCTRL
siPTPN2 48kDa
siPTPN2 45kDa
Control +
Control-
PTPN2 45kDa
PTPN2 48kDa
GAPDH
C)
48 kDa PTPN2 mRNA
45 kDa PTPN2 mRNA
PTPN2 mRNA levels (Normalized ratio over siCTRL)
D)
PTPN2:
48kDa
45kDa
Vinculin
E)
siCTRL
siPTPN2 48kDa
siPTPN2 45kDa
PTPN2 protein levels (Normalized ratio over NT)
48 kDa isoform
45 kDa isoform
F)
Ratio 48 kDa isoform over 45 kDa isoform protein levels
G)
PTPN1
Actin
H)
NT
siCTLR
siPTPN2 48kDa
siPTPN2 45kDa
PTPN1 protein levels (Normalized ratio over NT)

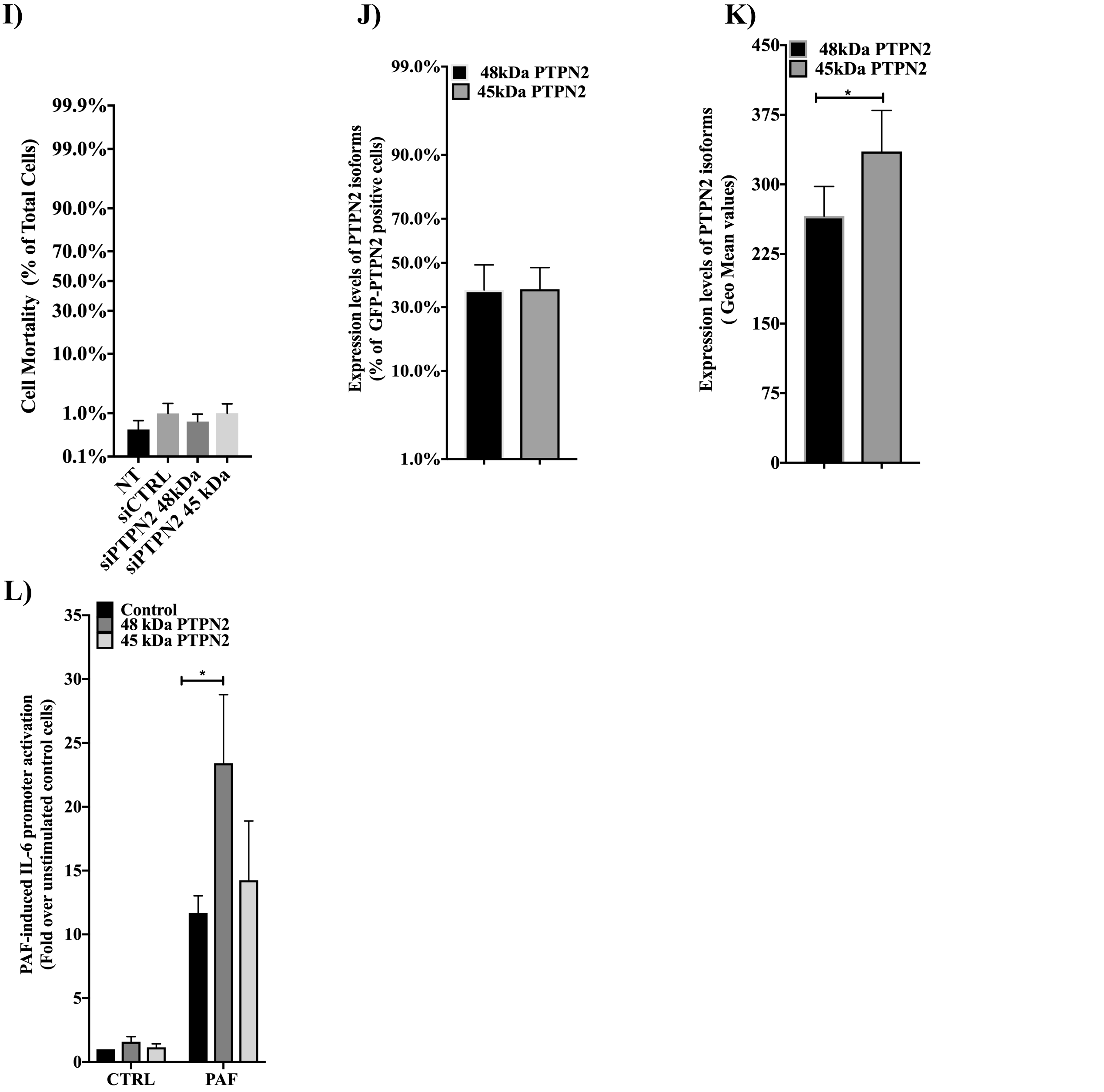

S-1 Figure. siRNAs against PTPN2 are isoform specific. A-G) iMo-DCs were not transfected **(NT)** or transfected with control siRNAs **(siCTRL)** or siRNAs against isoforms of PTPN2 **(siPTPN2 48kDa or 45kDa)** at day 4 and 5 before being collected at day 7 for experiments. **A-G)** iMo-DCs were lysed in Trizol and RNA was extracted and converted to cDNA. 45kDa PTPN2,48kDa PTPN2 and GAPDH were amplified by **A)** real-time PCR or **B-C)** PCR, loaded onto agarose gels and quantified. Representative gels and compilations of at least 4 independent experiments are shown in **B) and A) &C)** respectively. The data presented are mean±S.E.M. **A-C)** Significance was established with the Holm-Sidak's one-way analysis of variance. *p<0.05. **D-I)** iMo-DCs were starved for 5h in RPM1+0.2% BSA, **D-H)** then lysed. Whole cell lysates were separated by SDS-PAGE, transferred to nitrocellulose membranes and blotted overnight with Abs against **D)&F)** vinculin and PTPN2 or **G)&H)** actin and PTPN1. Representative blots and compilation of 4-5 independent experiments are shown. The data presented are mean±S.E.M of normalized protein levels over normalized protein levels of NT cells for **E)** 48 kDa, 45 isoform or **H)** PTPN1protein levels or **F)** ratio of 48 kDa isoform normalized protein levels over 45 kDa normalized protein levels. Significance was established with theTurkey's one-way analysis of variance. *p<0.05. **I)** Mortality was assayed by Trypan Blue staining. The data presented are mean±S.E.M of the % of Trypan-blue-positive iMo-DCs over total iMo-DCs for at least 6 independent experiments. **J)&K)** HEK-PAFR were transiently transfected with 48kDa or 45kDa PTPN2 tagged with GFP or left untransfected. Expression levels were determined by flow cytometry using untransfected cells for determination of basal fluorescence. The data presented are mean±S.E.M of **G)** the % of GFP-positive cells and of **H)** the Geo Mean value for 7 independent experiments performed in triplicate. Significance was established with paired Student's t-test. **L)** HEK-PAFR were transiently co-transfected with the hIL-6-luc, control vector (pcDNA3) or phosphatase constructs. Cells were incubated overnight in DMEM-0.2% BSA and stimulated with PAF (100nM) or its vehicle for 6h and luciferase activity was measured. The data presented are a mean±S.E.M of 7-14 independent experiments performed in triplicate. Significance was established with two-way ANOVA with Sidak post-test. *p<0.05.

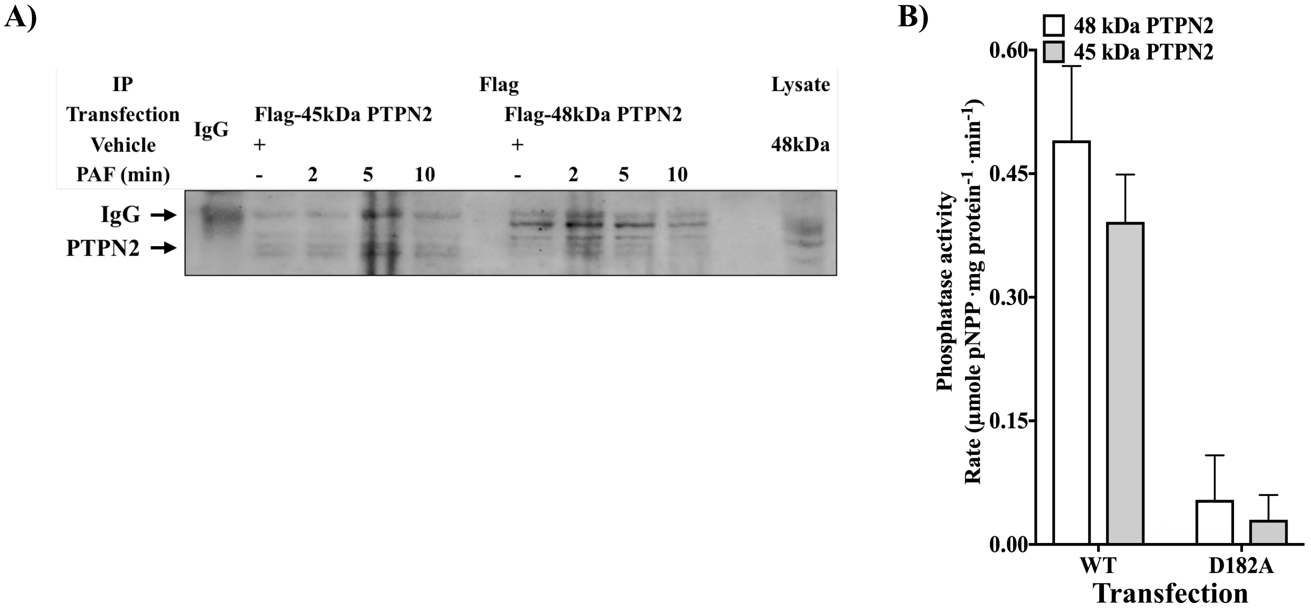

S-2 Figure. PTPN2 basal phosphatase activity. HEK-PAFR were transfected with Flag-tagged WT or D182A PTPN2 constructs, starved overnight in DMEM 0.2% BSA before being lysed. Immunoprecipitated PTPN2 was incubated with pNPP in PTP assay buffer, OD was taken at different incubation times at 37°C. 4x Laemmli buffer was added. The whole reaction mixture was separated by SDS-PAGE, transferred onto nitrocellulose membrane and incubated overnight with anti-PTPN2 Abs. Normalization of phosphatase activity was done according to the relative levels of PTPN2 detected by Western Blot. **A)** Representative blot used for normalization of PTP assays. **B)** Graph represents the phosphatase activity determined by the hydrolysis rate of pNPP by Flag- WT or -D182A PTPN2 isoforms immunoprecipitated from HEK-PAFR. Data are mean±S.E.M for 3 independent experiments. Significance was established with paired two-way ANOVA with Dunnett post-test.

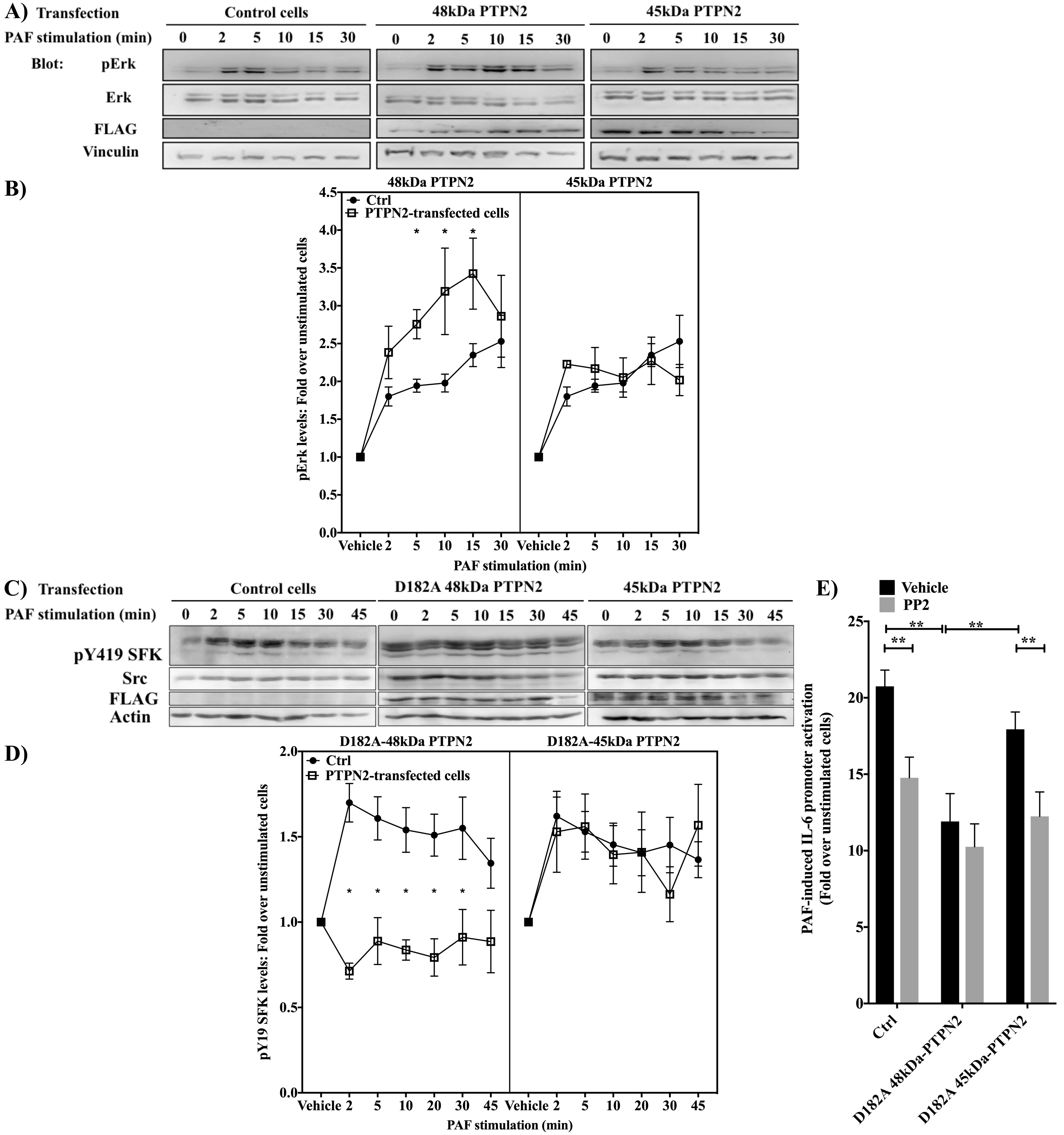

S-3 Figure. PTPN2 positively modulates PAF-induced Erk and SFK phosphorylation. **A-D)** HEK-PAFR were transiently transfected with **A&B)** WT or C&D) D182A 48kDa or 45kDa PTPN2 tagged with FLAG, or control vector (pcDNA3). Cells were incubated overnight in DMEM-0.2% BSA, then stimulated with PAF (100nM) or vehicle for indicated times. Whole cell lysates were separated by SDS-PAGE, transferred onto nitrocellulose membranes and blotted overnight with Abs against vinculin, Flag and **A)** pErk, and Erk or **C)** pY419 SFK and Src. Representative blots and compilations of at least 3 independent experiments are shown. **E)** HEK-PAFR were transiently co-transfected with hIL-6-luc, control vector (pcDNA3), or the D182A 48kDa or 45kDa PTPN2 constructs. Cells were incubated overnight in DMEM-0.2% BSA and pre-treated for 30 min in DMEM-0.2%BSA with DMSO as vehicle control or Src kinase family inhibitor PP2, 10nM. Stimulation with PAF (100nM) or vehicle was for 6h and luciferase activity was then measured. **A-E)** Data presented are mean±S.E.M. Significance was established with two-way ANOVA with Sidak post-test. *p<0.05, **p<0.01.

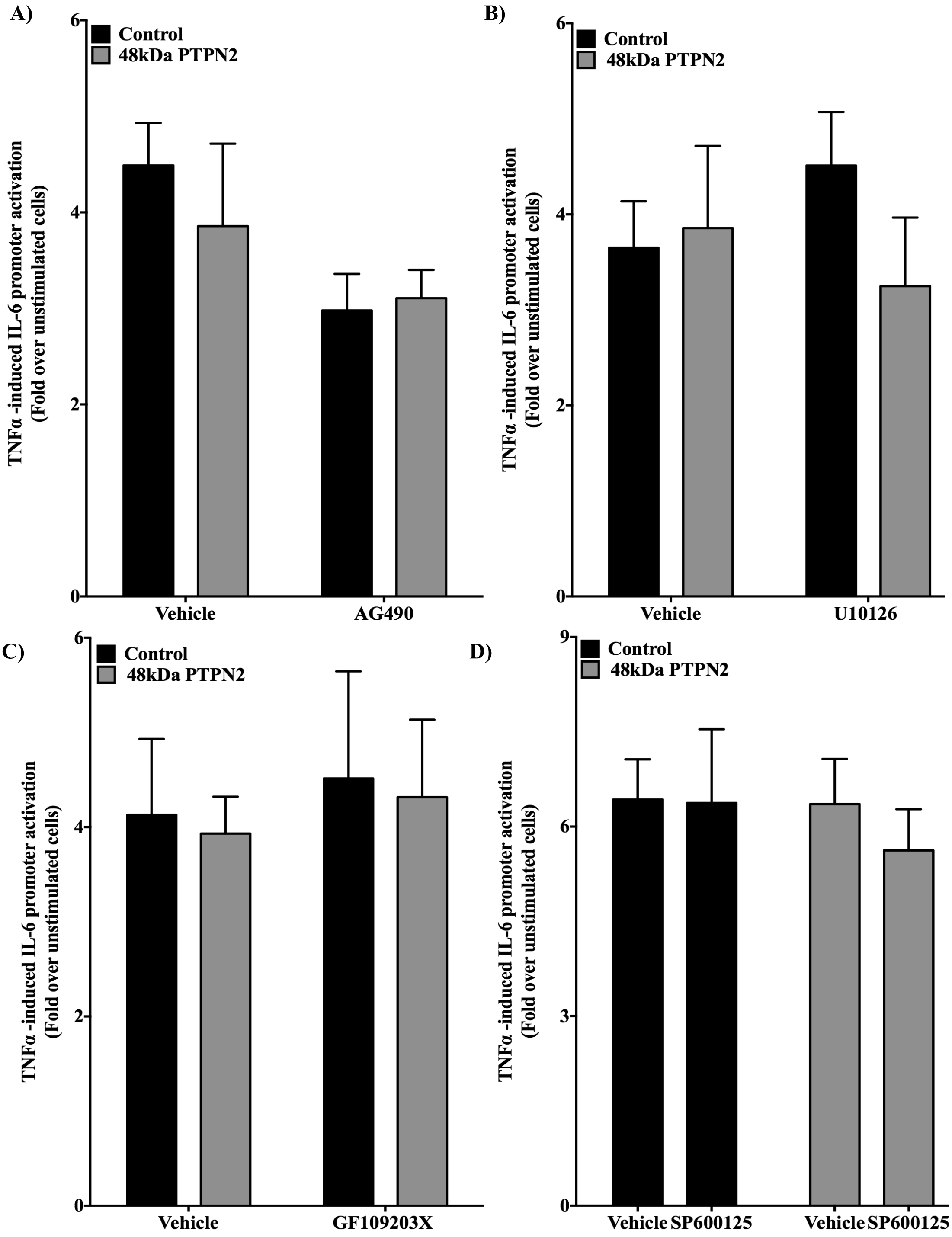

S-4 Figure. 48kDa PTPN2 and multiple kinase pharmacological inhibitors do not modulate TNFα-induced IL-6 promoter activity. HEK-PAFR were transiently co-transfected with the hIL-6-luc, the control vector pcDNA3 or the 48 kDa PTPN2 constructs. Cells were incubated overnight in DMEM-0.2% BSA and **A) to D)** pre-treated for 30 min in DMEM-0.2% BSA with DMSO as vehicle control or **A)** Jak2 inhibitor AG490 5µM; **B)** MEK 1 and 2 inhibitor U-0126, 15µM; **C)** pan PKCs inhibitor GF109203x 2 µM or **D)** JNK inhibitor SP600125, 5µM. Stimulation was done with TNFα (20 ng/ml) or vehicle for 6h and luciferase activity was measured. The data presented are mean±S.E.M of at least 4 independent experiments performed in triplicate.

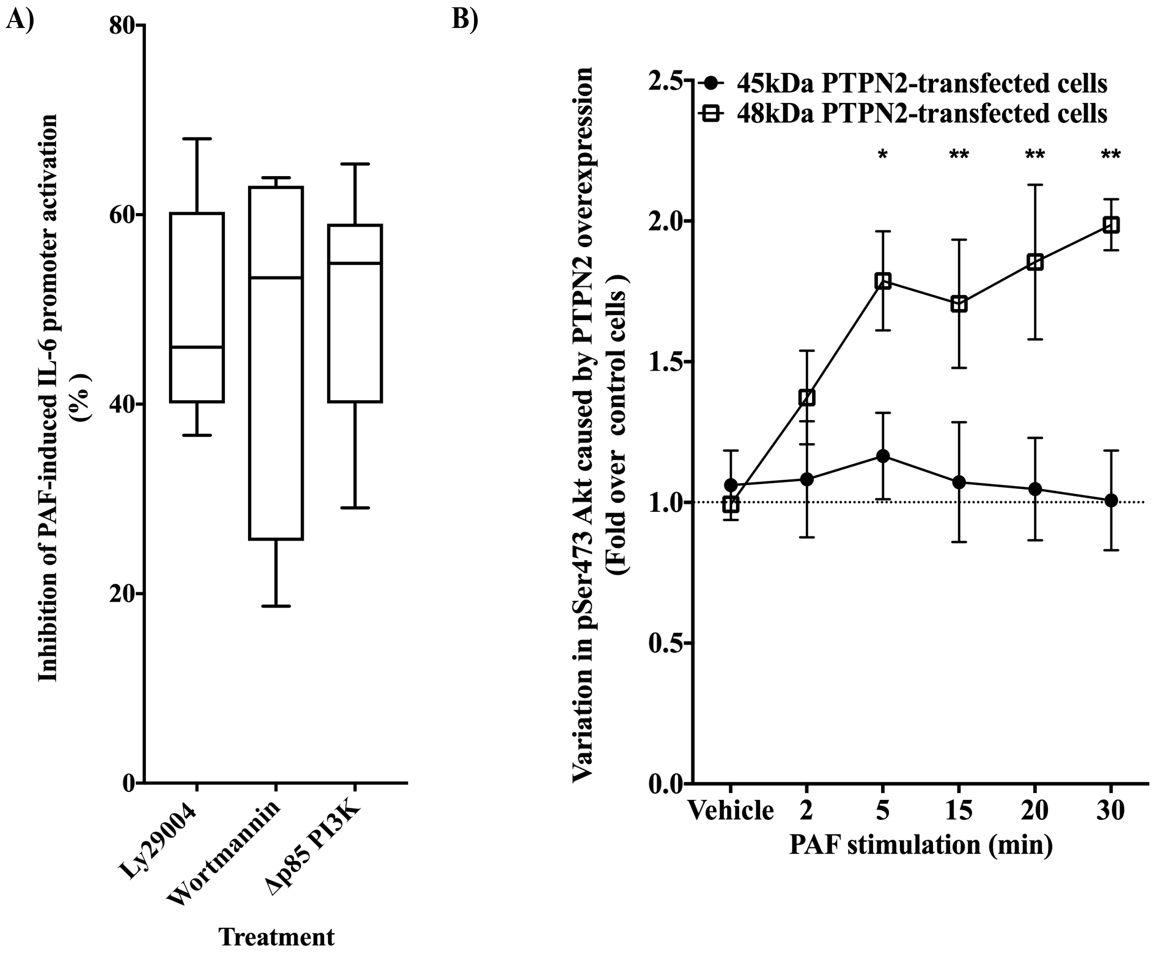

S-5 Figure. 48kDa PTPN2 and the PI3K/Akt pathway A) HEK-PAFR were transiently co-transfected with the IL-6 promoter coupled to luciferase. Cells were incubated overnight in DMEM-0.2% BSA and stimulated with PAF (100nM), after pre-treatment for 20 min in DMEM-0.2% BSA with DMSO as vehicle control or the PI3K inhibitors, LY294002 (10µM) and Wortmanin (100nM) or cells were co-transfected with Δp85 PI3K subunit constructs. Cells were incubated overnight in DMEM-0.2% BSA and stimulated with PAF (100nM) or vehicle for 6h and luciferase activity was measured. The data presented are mean±S.E.M of % inhibition caused by transfection of the dominant negative subunit or inhibitor treatment, for at least 4 experiments performed in triplicate. **B)** HEK-PAFR were transiently transfected with 48kDa or 45kDa PTPN2 cDNAs or control vector (pcDNA3). Cells were incubated overnight in DMEM-0.2% BSA, then stimulated with PAF (100nM) or vehicle for indicated times. Stimulation was stopped on ice, cells were scraped and lysed. Whole cell lysates were separated by SDS-PAGE, transferred to nitrocellulose membranes and blotted overnight with anti-pSer473Akt, anti-vinculin, anti-Flag and anti-Akt antibodies. Compilations of at least 4 independent experiments are shown. Percentages were determined by the ratio of pSer473Akt levels for PTPN2-overexpressing cells, normalized to Akt levels, over normalized pSer473Akt levels of control cells stimulated for the same time. The data presented are a mean±S.E.M. Significance was established with two-way ANOVA with Sidak post-test. *p<0.05, **p<0.01

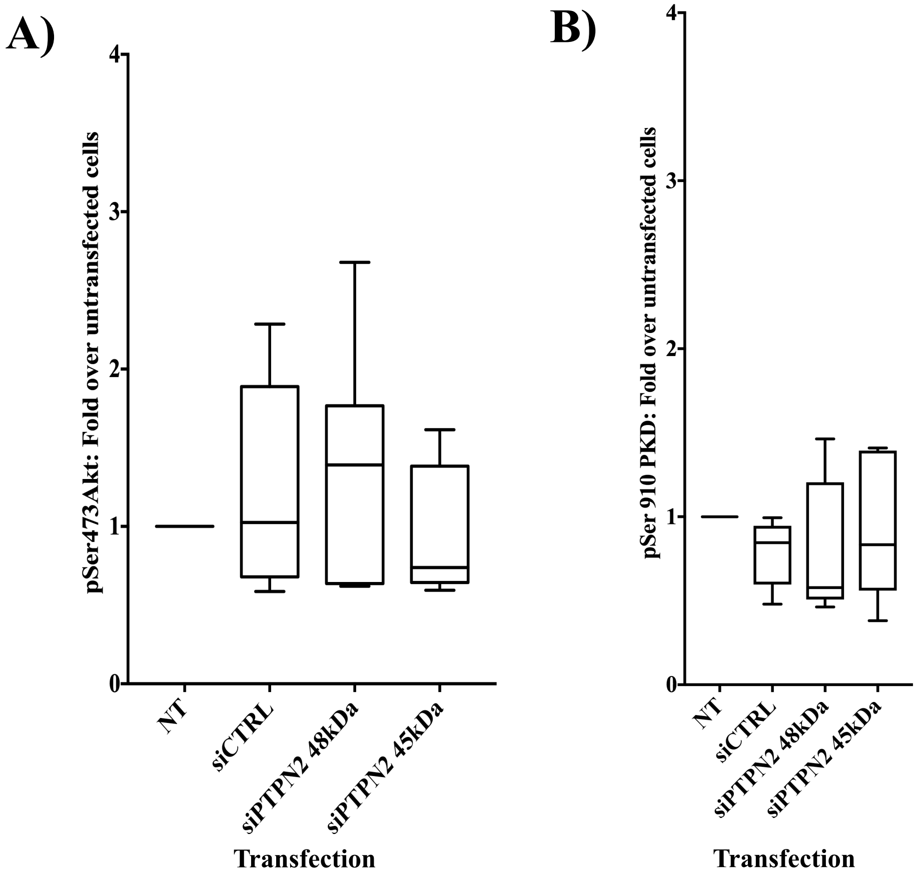

S-6 Figure. 48 kDa PTPN2 does not modulate basal PKD or Akt activation in iMoDCs. iMo-DCs were transfected with control siRNAs (siCTRL) or siRNAs against isoforms of PTPN2 (siPTPN2 48kDa or 45kDa) on day 4 and 5 before being collected on day 7 for experiments. iMo-DCs were starved for 5h in RPM1+0.2% BSA, stimulated with EtOH (PAF's vehicle) for 70min, centrifuged and lysed. Whole cell lysates were separated by SDS-PAGE, transferred to nitrocellulose membranes and blotted overnight with Abs against **A)** pSer473 Akt, Akt and vinculin/actin or **B)** pSer910PKD, PKD and actin. Compilation of at least 3 independent experiments are shown. The data presented are in a whisker and box graph (Min to Max).
